# The burden and trends of infectious diseases among children aged 14 and below in China from 1990 to 2021: a systematic analysis from the 2021 global burden of disease study

**DOI:** 10.3389/fpubh.2025.1541751

**Published:** 2025-05-09

**Authors:** Chun Guo Miao, Gui Rong Le, Si Yan Miao, Zhi Xin Liu

**Affiliations:** ^1^Department of Pediatrics, The People’s Hospital of Dongxiang, Fuzhou, China; ^2^Jiangxi Medical College, Nanchang University, Nanchang, China

**Keywords:** children, infectious diseases, global burden of disease (GBD), age-specific burden, HIV/AIDS

## Abstract

**Background:**

Infectious diseases remain one of the leading causes of death among children worldwide. This study aims to analyze the burden and trends of infectious diseases among children aged 0–14 years in China from 1990 to 2021, and evaluate their gender- and age-specific impacts.

**Methods:**

This study utilizes data from the Global Burden of Disease (GBD) 2021 to analyze mortality, incidence, disability-adjusted life years (DALYs), age-standardized mortality rates (ASMR), age-standardized incidence rates (ASIR), and age-standardized DALY rates (ASDR) for infectious diseases in Chinese children. Statistical analysis was performed using R and ggplot2.

**Results:**

From 1990 to 2021, China observed substantial declines in pediatric infectious disease burdens. Acute hepatitis mortality decreased from 7,349 deaths (95% UI:5,987–9,059) to 87 (EAPC: −13.78), with a fivefold reduction in ASMR. Enteric infections exhibited the steepest decline: incidence dropped by 86% (EAPC: −6.72), and ASDR fell from 2,257 to 67/100,000. HIV/AIDS deaths rose from 62 to 555 (EAPC:8.28), though post-2018 declines emerged. By 2021, lower respiratory infections remained the leading cause of death (ASMR:5.11/100,000), while upper respiratory infections had the highest incidence. Females showed faster reductions in enteric (EAPC: −7.44 vs. −6.20) and lower respiratory infections (EAPC: −10.39 vs. −9.76). Children under 5 faced the highest burden, particularly for lower respiratory infections (ASMR:13.02/100,000).

**Conclusion:**

The overall burden of infectious diseases among children in China has declined, especially for enteric infections and acute hepatitis. The burden of pediatric HIV/AIDS has also decreased in recent years, though adolescent HIV/AIDS education remains a key area of concern. Children under 5 continue to represent the highest burden group. While China’s infectious disease control measures and immunization programs have played a vital role, further strengthening policies to address ongoing challenges is essential for effectively reducing the burden of infectious diseases and achieving the Healthy China 2030 goals.

## Introduction

Infectious diseases remain a significant global public health issue, and their control and prevention continue to be key objectives today. A substantial portion of the mortality risk faced by children during their growth and development stems from infectious diseases (1, 2). It is estimated that approximately 3 million children die from infectious diseases each year (3). Reducing these deaths is crucial. According to data from the National Bureau of Statistics of China, the country’s natural population growth rate has been on a downward trend in recent years (4). Recent studies also indicate a continued decline in China’s total fertility rate (5), with policy interventions possibly failing to reverse this trend (6). In this challenging context, protecting the precious lives of children, actively preventing diseases, and reducing mortality rates remain critical issues.

Thanks to initiatives such as the Integrated Management of Childhood Illness (IMCI) and the UNICEF Child Survival Strategy, the global burden and mortality rate of infectious diseases among children under the age of 5 have significantly decreased, with similar trends observed in China (7). However, disparities in regional development and economic inequality persist, and the threat of infectious diseases remains, particularly in rural areas (8). There is still a lack of up-to-date and detailed epidemiological analysis of the infectious disease burden among children aged 0–14 in China. This study aims to provide a comprehensive analysis of the burden of infectious diseases among Chinese children, categorized by age and gender, using the Global Burden of Disease (GBD) database.

The Global Burden of Disease (GBD) database, led by the Institute for Health Metrics and Evaluation (IHME) at the University of Washington, is a global health impact database covering diseases, injuries, and risk factors. In this study, we utilized the latest GBD 2021 data to analyze the burden of infectious diseases among children in China. This research, based on the most recent statistical data, provides valuable insights into the current morbidity and mortality burden of infectious diseases in Chinese children, as well as their trends over time. The findings will offer important references for evaluating the effectiveness of relevant policies. Additionally, analyzing China-specific data will contribute to targeted actions and ongoing monitoring.

## Materials and methods

### Database version

The 2021 version of the Global Burden of Disease (GBD) database^1^ includes data on the severity of diseases, injuries, and risk factors across different ages, sexes, and time periods for 204 countries and regions worldwide. Nearly 10,000 researchers from 160 countries have contributed to the updates of this database. Disease data from 1990 to 2021 were obtained from this resource. Since the database is publicly available, the data downloaded does not contain any personal information and does not require ethical approval. This study adheres to the Guidelines for Accurate and Transparent Health Assessment Reporting (GARGE) (9).

### Definitions

The GBD 2021 includes 459 health outcomes and risk factors, which are categorized into three major groups: infectious, maternal, neonatal, and nutritional diseases; non-communicable diseases; and injuries. For this study, we included all infectious diseases under the first group, except for COVID-19 (from 1990 to 2019) and other unspecified infectious diseases for children aged 0–14 that are not recorded in the GBD 2021 data. This analysis therefore excludes these conditions. The diseases included are HIV/AIDS, sexually transmitted infections excluding HIV, tuberculosis, upper respiratory infections, lower respiratory infections, otitis media (International Classification of Diseases, 10th Revision: H65–H75.83), enteric infections, neglected tropical diseases and malaria, acute hepatitis, and other infectious diseases. We define children as those receiving pediatric services, including infants (under 5 years old), younger children (5–9 years old), and older children (10–14 years old) (10).

This study focuses on the burden of infectious diseases and their temporal trends in children. The burden indicators primarily include the number of deaths, incidence, disability-adjusted life years (DALYs), age-standardized mortality rate (ASMR), age-standardized incidence rate (ASIR), and age-standardized DALY rate (ASDR). Age-standardized rates are calculated based on the standard population age structure to eliminate the effects of population age composition. DALYs represent the total health years lost from illness to death, incorporating both the quantity and quality of life. Temporal trend indicators include the estimated annual percentage change (EAPC). If the EAPC and its 95% confidence interval (CI) are both positive, it indicates an increasing trend, while a negative EAPC suggests a decreasing trend; other situations indicate relative stability.

### Statistical methods

All statistical analyses and data visualizations were conducted using R (version 4.3.2) and ggplot2. Descriptive statistics were performed for all key variables, with results presented as means and 95% uncertainty intervals (UI). The calculation of the Estimated Annual Percentage Change (EAPC) and its 95% confidence interval (95% CI) follows the formula: 100 × (exp(*β*)-1). Where β represents the annual change in the natural logarithms of the age-standardized mortality rate (ASMR) and the age-standardized incidence rate (ASIR) (11, 12).

## Results

### Overall burden

**Figure 1:**
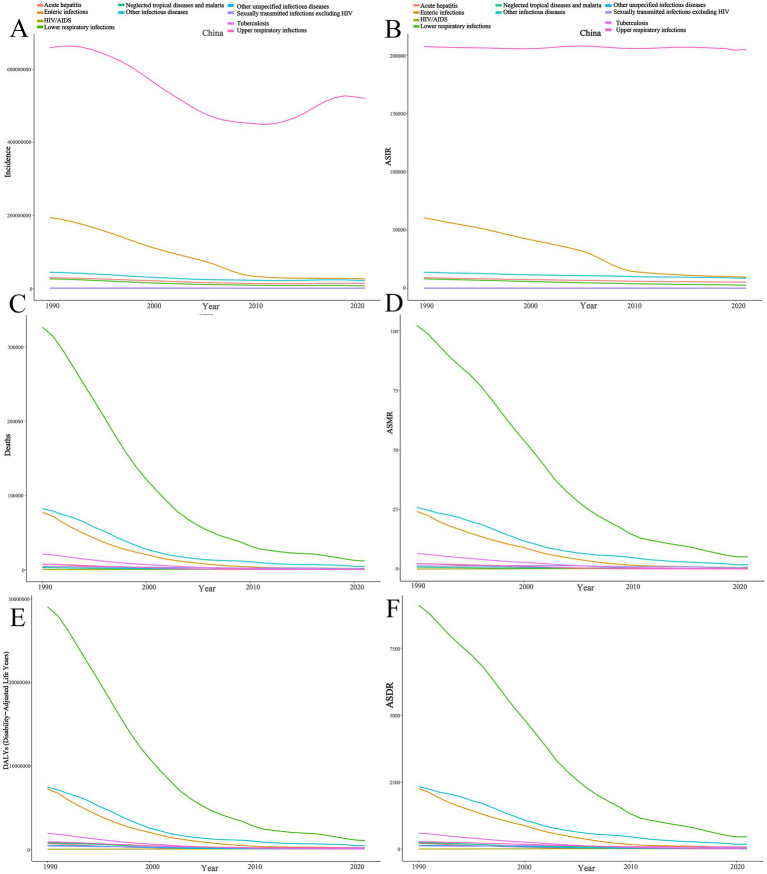
Trends of infectious diseases among children in China from 1990 to 2021. **(A)** Number of cases; **(B)** Age-standardized incidence rate; **(C)** Number of deaths; **(D)** Age-standardized mortality rate; **(E)** Number of DALYs; **(F)** Age-standardized DALYs rate.

**Figure 2:**
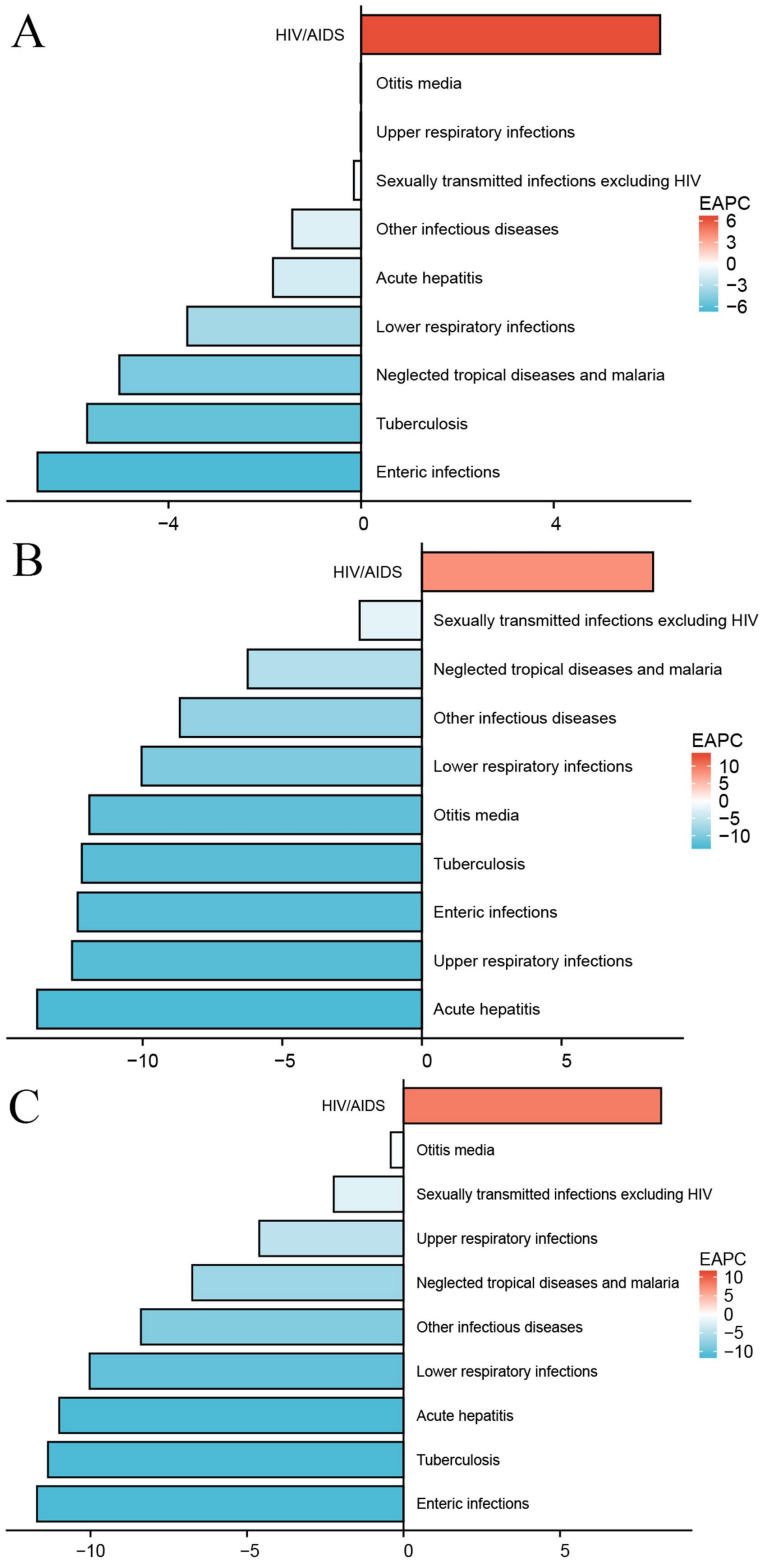
Estimated annual percentage change graph of infectious diseases among Chinese children. **(A)** Age-standardized incidence rate; **(B)** Age-standardized mortality rate; **(C)** Age-standardized DALYs rate.

From 1990 to 2021, the overall burden of infectious diseases among children in China showed a declining trend (Figure 1). In terms of incidence, with the exception of HIV/AIDS, which showed an increasing trend, and upper respiratory infections, which remained relatively stable, the burden of all other diseases decreased significantly (Figure 2A). The number of new HIV cases in 2021 (Supplementary Table 1) increased by approximately five times compared to 1990 (EAPC: 6.21, 95% CI: 4.77–7.66), with the age-standardized incidence rate (ASIR) increasing by about six times (Table 1). Among the diseases on the decline, the burden of enteric infections decreased the most rapidly, with the incidence in 2021 (Supplementary Table 1) being approximately 86% lower than 30 years ago (EAPC: −6.72, 95% UI: −7.24 to −6.18).

**Table 1:** Age-standardized prevalence rates of infectious diseases among children in China in 1990 and 2021.

Incidence (95% UI)			
		ASR in 1990 (per 100,000)	ASR in 2021 (per 100,000)
Acute hepatitis	Both	9114.88 (8150.80, 10104.75)	5326.58 (4689.72, 5986.28)
	Male	9349.05 (8294.71, 10514.77)	5332.79 (4686.57, 5992.24)
	Female	8860.24 (7917.99, 9958.62)	5319.60 (4672.74, 5964.97)
Enteric infections	Both	60877.38 (44236.14, 81984.66)	10005.03 (6465.37, 14331.85)
	Male	61977.54 (45030.96, 83131.63)	11624.31 (7506.92, 16638.77)
	Female	59648.04 (43030.03, 80356.43)	8149.74 (5253.10, 11674.99)
HIV/AIDS	Both	0.07 (0.03, 0.11)	0.45 (0.21,0.95)
	Male	0.07 (0.03, 0.11)	0.47 (0.21, 0.98)
	Female	0.07 (0.03, 0.11)	0.44 (0.21, 0.92)
Lower respiratory infections	Both	8112.76 (6919.89, 9564.68)	2520.56 (1973.16, 3196.71)
	Male	7787.84 (6646.59, 9146.33)	2415.83 (1901.33, 3062.55)
	Female	8480.65 (7250.17, 10033.21)	2641.02 (2063.50, 3353.99)
Neglected tropical diseases and malaria	Both	16.20 (13.37, 23.14)	2.92 (1.26, 7.31)
	Male	6.60 (13.72, 23.62)	2.90 (1.20, 7.82)
	Female	15.77 (12.93, 22.63)	2.94 (1.03, 8.68)
Other infectious diseases	Both	13693.15 (12474.43, 14914.27)	8630.69 (7765.15, 9531.80)
	Male	13853.55 (12515.06, 15210.83)	8635.13 (7764.34, 9567.03)
	Female	13522.05 (12317.28, 14785.78)	8626.16 (7756.29, 9485.00)
Sexually transmitted infections excluding HIV	Both	244.32 (143.08, 401.32)	228.38 (135.71, 376.64)
	Male	248.66 (140.22, 423.47)	229.58 (130.04, 392.66)
	Female	239.68 (144.77, 383.39)	227.00 (139.94, 353.65)
Tuberculosis	Both	53.18 (36.15, 73.70)	9.47 (6.67, 13.15)
	Male	50.52 (34.93, 69.74)	8.80 (6.19, 12.18)
	Female	56.08 (38.01, 78.39)	10.24 (7.20, 14.27)
Upper respiratory infections	Both	207307.78 (151211.61, 270357.48)	204933.59 (148251.54, 269026.79)
	Male	211444.19 (154859.03, 275916.44)	207658.24 (149914.27, 272150.27)
	Female	202729.42 (147179.65, 264399.73)	201806.33 (145698.06, 265860.63)
Otitis media	Both	10673.78 (6217.43, 17114.20)	10528.49 (5984.83, 17053.87)
	Male	10005.48 (5854.37, 16073.96)	9876.23 (5580.43, 16128.47)
	Female	11413.47 (6548.04, 18587.68)	11277.43 (6319.30, 18452.08)

Regarding mortality and DALYs, with the exception of HIV, which showed a clear upward trend, the burden of all other infectious diseases significantly decreased (Figures 2B,C). Acute hepatitis showed the fastest decline. In 1990, the number of deaths from acute hepatitis (Supplementary Table 2) was approximately 7,349 (95% UI: 5,987–9,059), but by 2021, it had dramatically decreased to 87 (95% UI: 61–122), with the age-standardized mortality rate (ASMR) (Table 2) decreasing by about five times (EAPC: −13.78, 95% CI: −14.43 to −13.11). In contrast, HIV-related deaths increased, with 62 deaths (95% UI: 18–109) in 1990 and 555 deaths (95% UI: 274–1,108) in 2021, corresponding to a nearly 12-fold increase in the ASMR (Table 2) (EAPC: 8.28, 95% CI: 6.94–9.62).

**Table 2:** Age-standardized mortality rates of infectious diseases among children in China in 1990 and 2021.

Death (95% UI)			
		ASR in 1990 (per 100,000)	ASR in 2021 (per 100,000)
Acute hepatitis	Both	2.30 (1.88, 2.84)	0.04 (0.03, 0.05)
	Male	2.19 (1.72, 2.78)	0.03 (0.02, 0.04)
	Female	2.40 (1.90, 3.09)	0.05 (0.03, 0.07)
Enteric infections	Both	24.17 (17.68, 30.85)	0.57 (0.41, 0.80)
	Male	23.79 (14.74, 33.49)	0.57 (0.36, 0.87)
	Female	24.61 (18.73, 31.19)	0.58 (0.40, 0.80)
HIV/AIDS	Both	0.02 (0.01, 0.03)	0.24 (0.12, 0.47)
	Male	0.02 (0.01, 0.03)	0.23 (0.11, 0.47)
	Female	0.02 (0.01, 0.03)	0.24 (0.12, 0.48)
Lower respiratory infections	Both	102.28 (87.15, 119.54)	5.11 (4.07, 6.29)
	Male	106.38 (88.94, 124.43)	5.77 (4.54, 7.13)
	Female	97.69 (82.87, 114.98)	4.35 (3.38, 5.46)
Neglected tropical diseases and malaria	Both	0.81 (0.51, 1.76)	0.09 (0.04, 0.19)
	Male	0.89 (0.52, 2.13)	0.11 (0.05, 0.25)
	Female	0.71 (0.47, 1.46)	0.07 (0.03, 0.14)
Other infectious diseases	Both	25.81 (16.96, 38.16)	1.88 (1.34, 2.75)
	Male	27.34 (17.66, 39.46)	2.03 (1.41, 2.97)
	Female	24.09 (15.45, 38.27)	1.72 (1.17, 2.57)
Sexually transmitted infections excluding HIV	Both	1.34 (0.47, 2.81)	0.84 (0.30, 1.86)
	Male	1.36 (0.48, 2.90)	0.86 (0.30, 1.87)
	Female	1.31 (0.46, 2.70)	0.82 (0.30, 1.86)
Tuberculosis	Both	6.49 (5.41, 7.65)	0.13 (0.10, 0.17)
	Male	6.09 (4.63, 7.85)	0.15 (0.11, 0.20)
	Female	6.93 (5.70, 8.31)	0.11 (0.08, 0.14)
Upper respiratory infections	Both	2.27 (0.49, 3.44)	0.06 (0.04, 0.14)
	Male	2.27 (0.40, 3.57)	0.08 (0.04, 0.18)
	Female	2.26 (0.42, 3.47)	0.05 (0.03, 0.12)
Otitis media	Both	0.00 (0.00, 0.01)	0.00 (0.00, 0.00)
	Male	0.00 (0.00, 0.01)	0.00 (0.00, 0.00)
	Female	0.00 (0.00, 0.01)	0.00 (0.00, 0.00)

The fastest decline in DALYs (Supplementary Table 3) was observed for enteric infections (EAPC: −11.71, 95% CI: −12.16 to −11.27), with the age-standardized DALY rate (ASDR) (Table 3) in 1990 at 2,257 (95% UI: 1,686–2,862) and dropping to 67 (95% UI: 50–89) in 2021. The ASDR for HIV in 2021 was 20.55/100,000 (95% UI: 10.04–41.38), an approximately 11-fold increase compared to 1990 (EAPC: 8.23, 95% CI: 6.90–9.58).

**Table 3:** Age-standardized DALYs rate of infectious diseases among children in China in 1990 and 2021.

DALYs (95% UI)			
		ASR in 1990 (per 100,000)	ASR in 2021 (per 100,000)
Acute hepatitis	Both	211.59 (172.82, 260.15)	8.69 (5.98, 12.42)
	Male	220.59 (174.64, 282.10)	9.53 (6.22, 14.16)
	Female	201.50 (159.27, 255.53)	7.72 (5.25, 11.11)
Enteric infections	Both	2257.18 (1686.09, 2861.65)	67.34 (49.64, 89.26)
	Male	2226.17 (1412.23, 3089.89)	69.68 (47.91, 98.06)
	Female	2292.41 (1760.58, 2877.59)	64.69 (47.51, 86.09)
HIV/AIDS	Both	1.72 (0.49, 3.00)	20.55 (10.04, 41.38)
	Male	1.72 (0.49, 3.00)	20.39 (9.97, 41.06)
	Female	1.72 (0.49, 3.01)	20.74 (10.12, 41.69)
Lower respiratory infections	Both	9099.27 (7760.29, 10624.83)	455.96 (363.74, 560.93)
	Male	9465.27 (7915.35, 11067.24)	513.91 (405.99, 634.10)
	Female	8688.41 (7380.01, 10223.23)	389.11 (303.13, 486.96)
Neglected tropical diseases and malaria	Both	244.70 (157.37, 365.16)	30.81 (20.37, 44.55)
	Male	269.44 (174.45, 410.23)	31.70 (20.39, 48.29)
	Female	217.45 (140.47, 320.11)	29.79 (20.08, 43.09)
Other infectious diseases	Both	2475.31 (1624.12, 3538.86)	197.70 (141.66, 283.19)
	Male	2167.50 (1402.14, 3411.41)	171.63 (121.53, 249.17)
	Female	2330.22 (1555.90, 3406.48)	185.59 (135.67, 263.22)
Sexually transmitted infections excluding HIV	Both	120.36 (42.82, 252.63)	75.72 (27.36, 167.80)
	Male	122.66 (43.53, 260.93)	77.22 (26.95, 168.26)
	Female	117.78 (41.54, 242.91)	74.00 (27.19, 167.46)
Tuberculosis	Both	593.77 (498.70, 699.96)	16.69 (13.46, 20.78)
	Male	556.85 (428.18, 712.81)	18.02 (14.14, 23.14)
	Female	635.18 (522.28, 760.07)	15.17 (11.68, 18.87)
Upper respiratory infections	Both	272.88 (108.36, 401.58)	78.59 (45.38, 127.69)
	Male	274.67 (101.39, 410.37)	80.66 (46.78, 130.27)
	Female	271.00 (95.02, 399.72)	76.20 (43.80, 125.56)
Otitis media	Both	46.71 (26.55, 75.04)	41.96 (23.65, 67.72)
	Male	9.69 (28.51, 80.13)	44.69 (25.56, 72.59)
	Female	43.50 (24.57, 70.46)	38.84 (21.44, 63.20)

**Figure 3:**
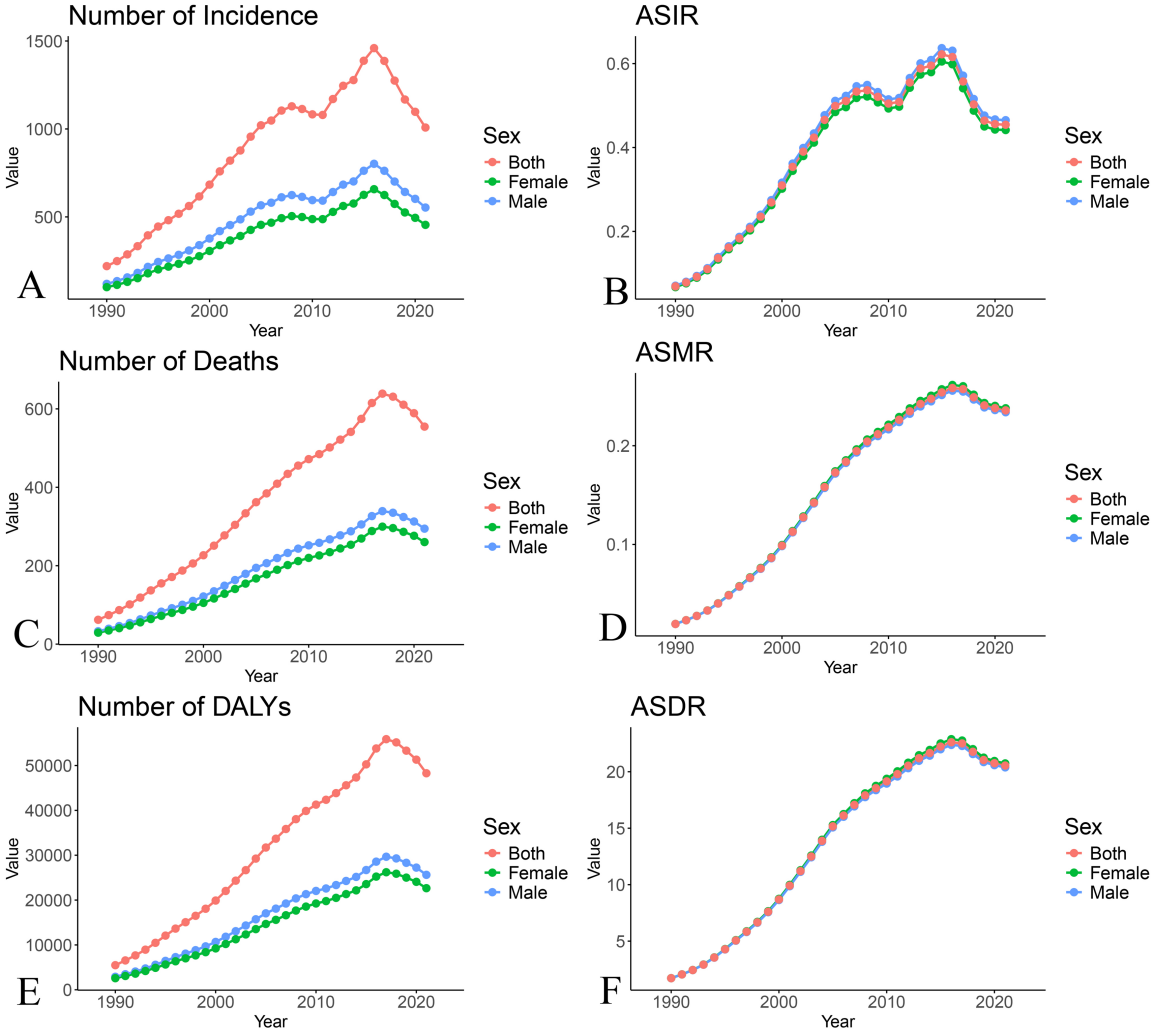
Trend chart of burden changes of HIV/AIDS among Chinese children from 1990 to 2021. **(A)** Number of cases; **(B)** Age-standardized incidence rate; **(C)** Number of deaths; **(D)** Age-standardized mortality rate; **(E)** Number of DALYs; **(F)** Age-standardized DALYs rate.

In 2021, the leading cause of infectious disease-related deaths among children in China was lower respiratory infections, while upper respiratory infections had the highest incidence. In conclusion, China has made significant progress in combating infectious diseases among children, but the rising burden of HIV remains a cause for concern (Figure 3).

### Gender-specific burden

In both 1990 and 2021, there were no significant gender differences in the burden of infectious diseases among children in China, though slight differences were observed for certain diseases and indicators. For example, in 2021, the age-standardized incidence rate (ASIR) for enteric infections was lower in females than in males (8,149/100,000 vs. 11,624/100,000), with a faster decline in females (EAPC: −7.44 vs. −6.20). Similarly, the age-standardized death rate (ASDR) for lower respiratory infections was lower in females (389/100,000 vs. 514/100,000), with a faster decline in females (EAPC: −10.39 vs. −9.76). In terms of age-standardized mortality rate (ASMR), females had slightly lower rates for lower respiratory infections, neglected tropical diseases, malaria, other infectious diseases, tuberculosis, and lower respiratory infections, while acute hepatitis was slightly higher in males. No significant age-related differences or epidemiological changes were observed between 1990 and 2021. For HIV in China, the number of cases, deaths, and DALYs were higher in males compared to females, though no noticeable differences were found in the ASIR, ASMR, or ASDR (Figure 3).

### Age-specific burden

We also analyzed the burden of infectious diseases among children of different age groups in China. Supplementary Table 4 presents the age-standardized incidence rate (ASIR) for infectious diseases in 1990 and 2021 across different age groups. The results show that acute hepatitis, enteric infections, HIV/AIDS, lower respiratory infections, other infectious diseases, otitis media, tuberculosis, and upper respiratory infections had the heaviest burden in children under 5 years old. In contrast, sexually transmitted diseases (excluding HIV) and neglected tropical diseases and malaria had the highest burden in the 10–14 age group. Regarding age-standardized mortality rate (ASMR) (Supplementary Table 5), the highest mortality rates for all included infectious diseases were observed in the under-5 age group. In 2021, most infectious disease-related ASMRs were at relatively low levels. However, it is noteworthy that the ASMR for lower respiratory infections in children under 5 years old was approximately 13.02/100,000, significantly higher than for other infectious diseases.

For age-standardized DALY rate (ASDR) (Supplementary Table 6), otitis media had the highest burden in the 5–9 age group in both 1990 and 2021. In 1990, neglected tropical diseases and malaria had the heaviest burden in children under 5 years old, but by 2021, this burden shifted to the 10–14 age group. Overall, children under 5 years old in China contributed a large proportion of the infectious disease burden, and infectious disease-related mortality in this age group has significantly decreased over the past few decades.

## Discussion

Globally, millions of children die from infectious diseases each year, though in China, the number of child deaths due to infectious diseases has been decreasing steadily. However, as a populous nation with imbalanced development and disparities in healthcare resource distribution, challenges still remain (13). Our study results show that the burden of infectious diseases in Chinese children has declined for almost all diseases, except HIV. Among these, the burden from enteric infections and the associated DALYs have decreased most significantly. Diarrheal diseases, which cause severe dehydration and metabolic imbalances in children, are a major cause of child mortality (14), and China is one of the 15 countries with the highest burden of diarrheal disease globally. An epidemiological study involving 31 provinces and over 200 hospitals in China found that the most common pathogen for children under 5 years old was rotavirus A, whereas norovirus predominated in older age groups (15). In 2000, the World Health Organization (WHO) issued a report advocating for rotavirus vaccine research, and a recent meta-analysis demonstrated that the rotavirus vaccine significantly reduces the incidence and hospitalization rates of rotavirus gastroenteritis in children (16). WHO’s advocacy and the widespread availability of the rotavirus vaccine may be important factors contributing to the decline in diarrheal disease burden. Furthermore, studies suggest that rotavirus vaccines have significant potential to be included as a second-priority vaccine in China’s national immunization program (17).

Our findings align with previous research showing that lower respiratory infections were the leading cause of infectious disease-related deaths in Chinese children (18). In 1990, the age-standardized mortality rate (ASMR) was approximately 102/100,000, but by 2021, it had significantly decreased to 5.11/100,000. The ASMR declined nearly 20-fold over the past 30 years, yet lower respiratory infections remain the leading cause of death. The ASMR for most other infectious diseases was below 1/100,000, and DALYs were also at relatively low levels. Achieving such improvements in a country with a population of over 1.4 billion is a remarkable achievement. The Chinese government has developed and continuously refined its infectious disease prevention and control system over the past decades, with funding for disease control increasing fivefold (19). The China CDC is now able to receive detailed reports on infectious diseases within hours. Economic improvements, along with enhanced child healthcare and nutrition, have also played a significant role. Since the early 21st century, per capita disposable income in rural and urban China has increased, with a rising percentage of children benefiting from systematic health management and a decreasing prevalence of child malnutrition. These improvements have contributed to the gradual reduction in the incidence and mortality of infectious diseases among children (20).

The Ministry of Health (now the National Health Commission) launched the national immunization program in 1978, achieving significant milestones over the past 40 years (21). Other infectious diseases included in the GBD 2021 category mainly refer to vaccine-preventable diseases such as encephalitis, tetanus, and diseases covered by the DTP vaccine. Our results also show a marked decline in these diseases over the past 30 years. Over the past three decades, China has significantly strengthened the surveillance of Japanese encephalitis (JE) and meningococcal meningitis in endemic regions (e.g., rural areas of Yunnan and Sichuan provinces) through the National Notifiable Infectious Disease Reporting System managed by the China Center for Disease Control and Prevention. Vector control measures, including the deployment of insecticide-treated bed nets and larval source management, have been rigorously implemented in high-risk zones. The introduction of the live attenuated JE vaccine into the National Immunization Program (NIP) in 2007 has achieved >90% coverage in endemic areas. Meningococcal vaccines (polysaccharide and conjugate formulations) have been routinely administered since the 1980s (22). Future strategies should prioritize expanding conjugate meningococcal vaccine coverage to adolescents (currently limited to high-risk groups) and integrating JE vaccination into school health programs in non-endemic urban areas with migrant populations (23). The diphtheria-tetanus-pertussis vaccine, containing whole-cell pertussis (wP) and diphtheria-tetanus toxoids, has been included in the NIP since 1978. To address safety concerns and improve compliance, replacing wP with acellular pertussis (aP) vaccines and introducing maternal Tdap vaccination to protect neonates are critical next steps (24). Nationwide supplementary immunization activities (SIAs) targeting measles elimination, initiated in 2010, have achieved 95% coverage. However, varicella remains excluded from the NIP, requiring out-of-pocket payment in most provinces. Mandatory varicella reporting in schools and childcare facilities has been enforced. Accelerating the integration of varicella vaccines into the NIP and addressing measles immunity gaps among migrant children through mobile vaccination units are essential measures (25, 26). Universal hepatitis B (HepB) vaccination for newborns, implemented in 1992, and the inclusion of the H2 strain live attenuated hepatitis A (HepA) vaccine in the NIP in 2008 represent major milestones. Future efforts should focus on subsidized vaccination campaigns in rural areas with low HepA coverage and systematic screening and treatment of HBV carriers among pregnant women to eliminate vertical transmission (27, 28). However, challenges persist in China’s national immunization program, including vaccine shortages and insufficient vaccine efficacy (29). The low profitability of vaccines has led to insufficient incentives for manufacturers, and low salaries for CDC staff have contributed to staff shortages (30). Additionally, the lack of a formal mechanism for the National Immunization Advisory Committee has hindered the optimization of the national immunization schedule, such as the inclusion of the Hib (*Haemophilus influenzae* type b) vaccine and the rotavirus vaccine. Economic disparities among different counties and cities also lead to inequalities in vaccine access. A unified, coordinated policy to address these challenges is crucial for the realization of the Healthy China 2030 initiative. To address these issues, the following strategies are proposed: Policy Integration: Expedite the inclusion of varicella, meningococcal conjugate, and maternal Tdap vaccines into the NIP; Public-Private Partnerships: Subsidize vaccine production through government-industry collaborations to enhance affordability and accessibility; Digital Innovation: Implement blockchain-based vaccine traceability systems to ensure supply chain integrity and combat counterfeit products; Community Engagement: Leverage social media platforms to disseminate evidence-based information, debunk vaccine myths, and cultivate public trust (31).

China has achieved remarkable progress in reducing the burden of tuberculosis (TB) and malaria among children over the past three decades, driven by evidence-based policies and sustained public health investments. The mortality of TB in children declining from 6.49 to 0.13 per 100,000. This success is attributed to a multi-pronged strategy: National TB Control Program (2001–2010): Prioritized early detection through free sputum smear microscopy and expanded access to directly observed therapy (DOTS). GeneXpert MTB/RIF Rollout: Rapid molecular diagnostics were introduced in 2011 to identify drug-resistant TB, reducing diagnostic delays from weeks to hours (32). High-Risk Population Screening: Mandatory TB screening in schools and migrant worker communities, particularly in high-burden regions (33, 34). BCG Vaccination: Universal neonatal BCG vaccination (coverage >99% since 1978) significantly reduced disseminated TB cases. China eliminated indigenous malaria transmission by 2017 and received WHO certification in 2021, with pediatric malaria mortality declining by 79% (0.81 to 0.09 per 100,000). Key interventions included: “1–3-7” Surveillance-Response Model. 1 day to report cases, 3 days to investigate, and 7 days to implement vector control (e.g., insecticide-treated bed nets, indoor residual spraying) (35, 36). Cross-Border Collaboration: Joint surveillance and rapid response mechanisms with neighboring countries (Myanmar, Laos, Vietnam) to prevent re-introduction (37). Mobile Health Units: Deployed in remote areas (Yunnan, Hainan) to deliver artemisinin-based combination therapy and rapid diagnostic tests. Despite these gains, disparities persist in rural healthcare access and drug-resistant TB management (38). Strengthening primary healthcare systems and integrating AI-driven surveillance tools will be critical to achieving the Healthy China 2030 targets.

The only increase in the burden of infectious diseases observed in our study was related to HIV/AIDS. The Estimated Annual Percentage Change (EAPC) is a composite measure that reflects the average trend over 30 years, and it is noteworthy that the burden of HIV/AIDS started to decline around 2018, after years of continuous increase. This trend is consistent with research by Qiao et al. (39). Although the overall burden of HIV/AIDS remains relatively low, with an ASIR of 0.45/100,000 and an ASMR of 0.24/100,000 in 2021, the majority of infections and deaths in children under 15 years old are due to mother-to-child transmission. Our findings suggest that the burden of HIV/AIDS in Chinese children has been largely controlled in recent years, which is consistent with the latest national survey and the findings of Dong et al. (40). Given the transmission route of HIV/AIDS, the situation suggests that China still faces challenges in preventing and controlling HIV/AIDS. While China introduced initiatives and policies targeting youth as early as 2015, an editorial on the rising number of HIV infections among Chinese students reported that the number of university students infected with HIV is increasing by 50% each year (41). This is coupled with a growing openness among young people towards sex, with 80% engaging in premarital sex and having multiple sexual partners, yet only about 50% receive sexual education. The lack of comprehensive sexual education may contribute to the increasing risk of HIV transmission. More proactive measures and international collaboration will be crucial in reducing HIV/AIDS incidence and mortality and minimizing the risk of mother-to-child transmission (42, 43). Our study did not observe widespread gender differences in the infectious disease burden in China. However, Yin et al. (44) found that female sex was a protective factor for survival time among children with HIV/AIDS. Our findings similarly show that the number of HIV-related deaths and DALYs was higher in males than in females.

Age-specific burden distribution indicates that most of the infectious disease burden in China is concentrated in children under 5 years of age, particularly for lower respiratory infections. The mortality burden for infectious diseases in children aged 0–14 years remains low, with most ASMRs under 0.1/100,000. Health policies focused on children under 5 years old remain a priority. However, for certain diseases, the burden in the over-5 age group remains substantial. For instance, acute hepatitis accounts for about 51.68% of the total burden in the 0–14 age group, and enteric infections account for about 60.12%. Addressing the infectious disease burden in these age groups would help further reduce the healthcare burden. However, national strategic planning and resource allocation for these age groups remain underdeveloped (45).

This study utilizes data from the GBD 2021, which demonstrates core advancements over the GBD 2019 in terms of improved data timeliness and accuracy. The GBD 2021 has extended the disease burden data to 2021, comprehensively covering the critical period of the COVID-19 pandemic. Furthermore, through optimized statistical models and expanded data sources, the GBD 2021 significantly enhances the reliability of its results. Additionally, the adoption of more rigorous data validation mechanisms in the GBD 2021 reduces data missingness bias at sub-provincial levels. These improvements not only strengthen the credibility of cross-temporal comparisons but also provide a more scientific benchmark for evaluating the dynamic progress toward the “Healthy China 2030” goals. This study analyzes the current burden of infectious diseases among Chinese children and the trends over the past 30 years. It provides age-specific and gender-specific data, offering valuable insights for policymakers. To achieve the Healthy China 2030 goals, a multi-pronged approach is essential: (1) Optimizing immunization programs by expanding the National Immunization Program, while transitioning to safer acellular pertussis formulations; (2) Strengthening digital surveillance through blockchain-based traceability systems and AI-driven diagnostics to enhance disease monitoring and vaccine supply chain integrity; (3) Ensuring equitable access via mobile vaccination units for migrant populations and subsidized rural vaccination campaigns; (4) Targeted disease control through adolescent HIV education, rapid TB diagnostics, and cross-border malaria prevention; (5) Health system strengthening by addressing workforce shortages and establishing a National Immunization Advisory Committee to guide policy updates; and (6) Community engagement leveraging social media to combat vaccine hesitancy and integrate school-based health initiatives. These strategies build on China’s successes—such as measles elimination, universal HepB vaccination, and malaria eradication via the “1–3-7” model—yet require sustained efforts to overcome persistent challenges, including rural–urban disparities and vaccine supply shortages. Our study also finds that the burden of HIV/AIDS in Chinese children has decreased in recent years, marking a shift from previous trends. Despite these advancements, persistent challenges require urgent attention. From a clinical perspective, the following priorities are critical: (1) strengthening early identification and standardized management protocols for lower respiratory infections in children under 5 years old, given their persistently high mortality burden (46, 47) (2) accelerating the inclusion of rotavirus vaccines into the National Immunization Program, supported by their pivotal role in reducing enteric infections; and (3) establishing hospital-based infectious disease surveillance systems, particularly in rural areas where diagnostic delays exacerbate clinical outcomes. These strategies, grounded in the study’s findings, align with the “Healthy China 2030” framework and emphasize data-driven interventions to optimize pediatric care.

However, this study has some limitations. The data from China may contain discrepancies or inaccuracies, which could affect the accuracy of disease burden estimates. Currently, GBD 2021 does not include provincial, municipal, or county-level data from China, nor does it account for seasonal or climatic factors, making regional and seasonality analyses impossible. The update of such data will be crucial for enhancing the applicability of future GBD estimates. Similarly, GBD 2021 lacks pathogen data for respiratory infections. We did not conduct time trend, age group, and gender analyses for all infectious disease subtypes. Moreover, the lack of comparative analyses with global, national, and regional data limits the broader applicability of the findings.

## Conclusion

The overall burden of infectious diseases among children in China has declined, especially for enteric infections and acute hepatitis. The burden of pediatric HIV/AIDS has also decreased in recent years, though adolescent HIV/AIDS education remains a key area of concern. Children under 5 continue to represent the highest burden group. While China’s infectious disease control measures and immunization programs have played a vital role, further strengthening policies to address ongoing challenges is essential for effectively reducing the burden of infectious diseases and achieving the Healthy China 2030 goals.

## Data Availability

The raw data supporting the conclusions of this article will be made available by the authors, without undue reservation.

## Footnotes

^1^ https://ghdx.healthdata.org/gbd-2021
