# Supplementary material for: The burden and trends of infectious diseases among children aged 14 and below in China from 1990 to 2021: a systematic analysis from the 2021 global burden of disease study
Source: Front Public Health. 2025 May 9;13:1541751. doi: 10.3389/fpubh.2025.1541751 (PMC12098070; doi:10.3389/fpubh.2025.1541751)
Supplement: Supplementary file 1 [file Table_1.DOCX]

Table 1: Incidence rate burden of infectious diseases among children in China

| Incidence (95% UI) | | | | | | |
| --- | --- | --- | --- | --- | --- | --- |
|  | | Cases in 1990 (million) | Cases in 2021(million) | ASR in 1990 (per 100 000) | ASR in 2021 (per 100 000) | EAPC (95% CI) |
| Acute hepatitis | Both | 29005214.03(25931321.77,32162516.17) | 13386369.89(11787231.01,15035595.52) | 9114.88(8150.80,10104.75) | 5326.58(4689.72,5986.28) | -1.83(-1.89,-1.77) |
|  | Male | 15606834.84(13844402.67,17553510.33) | 7161261.59(6295483.93,8043624.26) | 9349.05(8294.71,10514.77) | 5332.79(4686.57,5992.24) | -1.93(-2.00,-1.86) |
|  | Female | 13398379.19(11969232.26,15067702.89) | 6225108.30(5470332.20,6977002.29) | 8860.24(7917.99,9958.62) | 5319.60(4672.74,5964.97) | -1.72(-1.78,-1.66) |
| Enteric infections | Both | 193716277.11(140817135.69,260917085.24) | 25626018.75(16389215.60,36867172.64) | 60877.38(44236.14,81984.66) | 10005.03(6465.37,14331.85) | -6.72(-7.24,-6.18) |
|  | Male | 103627933.70(75406007.61,138885690.64) | 15899061.67(10164724.05,22847364.11) | 61977.54(45030.96,83131.63) | 11624.31(7506.92,16638.77) | -6.20(-6.68,-5.72) |
|  | Female | 90088343.41(64936751.46,121517840.41) | 9726957.07(6204591.70,14004386.60) | 59648.04(43030.03,80356.43) | 8149.74(5253.10,11674.99) | -7.44(-8.05,-6.83) |
| HIV/AIDS | Both | 220.23(106.76,356.00) | 1008.08(466.07,2116.42) | 0.07(0.03,0.11) | 0.45(0.21,0.95) | 6.21(4.77,7.66) |
|  | Male | 119.48(58.00,192.52) | 553.28(255.52,1161.24) | 0.07(0.03,0.11) | 0.47(0.21,0.98) | 6.22(4.78,7.67) |
|  | Female | 100.75(48.76,163.36) | 454.80(211.71,949.95) | 0.07(0.03,0.11) | 0.44(0.21,0.92) | 6.19(4.76,7.65) |
| Lower respiratory infections | Both | 25796974.90(22011041.90,30402299.51) | 6450158.67(5017957.67,8233847.03) | 8112.76(6919.89,9564.68) | 2520.56(1973.16,3196.71) | -3.61(-3.69,-3.53) |
|  | Male | 13042173.03(11140387.44,15303405.53) | 3307930.00(2588855.30,4221696.34) | 7787.84(6646.59,9146.33) | 2415.83(1901.33,3062.55) | -3.66(-3.74,-3.58) |
|  | Female | 12754801.87(10901716.66,15094200.76) | 3142228.67(2439513.61,4017378.60) | 8480.65(7250.17,10033.21) | 2641.02(2063.50,3353.99) | -3.55(-3.66,-3.44) |
| Neglected tropical diseases and malaria | Both | 51748.91(42686.27,73833.07) | 7776.98(3364.26,19438.55) | 16.20(13.37,23.14) | 2.92(1.26,7.31) | -5.02(-6.20,-3.83) |
|  | Male | 27600.56(22815.13,39278.57) | 4120.30(1710.24,11086.69) | 6.60(13.72,23.62) | 2.90(1.20,7.82) | -5.10(-6.21,-3.97) |
|  | Female | 24148.36(19790.04,34581.78) | 3656.68(1286.55,10761.54) | 15.77(12.93,22.63) | 2.94(1.03,8.68) | -4.95(-6.21,-3.67) |
| Other infectious diseases | Both | 43599288.67(39716228.88,47487242.84) | 20961363.49(18819177.57,23215325.46) | 13693.15(12474.43,14914.27) | 8630.69(7765.15,9531.80) | -1.43(-1.47,-1.39) |
|  | Male | 23199670.52(20961551.77,25467099.38) | 11222679.46(10068088.84,12468062.58) | 13853.55(12515.06,15210.83) | 8635.13(7764.34,9567.03) | -1.49(-1.53,-1.44) |
|  | Female | 20399618.14(18575846.76,22319263.52) | 9738684.03(8736853.12,10740519.46) | 13522.05(12317.28,14785.78) | 8626.16(7756.29,9485.00) | -1.37(-1.42,-1.33) |
| Sexually transmitted infections excluding HIV | Both | 796883.51(466640.68,1309018.75) | 625234.48(371196.48,1032012.14) | 244.32(143.08,401.32) | 228.38(135.71,376.64) | -0.15(-0.25,-0.05) |
|  | Male | 418938.17(236242.11,713429.85) | 335405.55(189757.88,574197.22) | 248.66(140.22,423.47) | 229.58(130.04,392.66) | -0.31(-0.37,-0.25) |
|  | Female | 377945.34(228264.76,604621.16) | 289828.93(178550.50,451829.05) | 239.68(144.77,383.39) | 227.00(139.94,353.65) | 0.02(-0.21,0.25) |
| Tuberculosis | Both | 169980.06(115539.55,235542.64) | 23905.61(16632.42,33496.75) | 53.18(36.15,73.70) | 9.47(6.67,13.15) | -5.69(-5.87,-5.51) |
|  | Male | 84549.39(58516.74,116607.22) | 11840.21(8221.72,16532.17) | 50.52(34.93,69.74) | 8.80(6.19,12.18) | -5.70(-5.88,-5.51) |
|  | Female | 85430.66(57818.93,119544.12) | 12065.40(8382.85,16972.55) | 56.08(38.01,78.39) | 10.24(7.20,14.27) | -5.67(-5.86,-5.49) |
| Upper respiratory infections | Both | 659009081.59(480884317.06,859348436.04) | 519930804.64(373818454.77,686077791.90) | 207307.78(151211.61,270357.48) | 204933.59(148251.54,269026.79) | -0.01(-0.03,0.00) |
|  | Male | 352121172.41(258172037.99,459150070.71) | 281262841.31(201854198.73,370508054.03) | 211444.19(154859.03,275916.44) | 207658.24(149914.27,272150.27) | -0.03(-0.05,-0.01) |
|  | Female | 306887909.18(222714279.78,400490504.26) | 238667963.34(171244691.56,316008168.83) | 202729.42(147179.65,264399.73) | 201806.33(145698.06,265860.63) | 0.00(-0.01,0.01) |
| Otitis media | Both | 33897330.12(19758077.25,54330107.09) | 26021342.06(14619414.11,42549725.53) | 10673.78(6217.43,17114.20) | 10528.49(5984.83,17053.87) | -0.01(-0.02,-0.00) |
|  | Male | 16695361.24(9790269.43,26789856.82) | 13040950.90(7274133.55,21457966.50) | 10005.48(5854.37,16073.96) | 9876.23(5580.43,16128.47) | -0.01(-0.02,0.00) |
|  | Female | 17201968.89(9862361.15,28027956.69) | 12980391.17(7201106.04,21437712.81) | 11413.47(6548.04,18587.68) | 11277.43(6319.30,18452.08) | -0.01(-0.02,0.00) |

Table 2: Burden of mortality from infectious diseases among children in China

|  |  | Deaths(95% UI) | | | | |
| --- | --- | --- | --- | --- | --- | --- |
|  |  | Cases in 1990 (million) | Cases in 2021(million) | ASR in 1990 (per 100 000) | ASR in 2021 (per 100 000) | EAPC (95% CI) |
|  |  |  |  |  |  |  |
| Acute hepatitis | Both | 7348.93(5987.18,9059.01) | 87.02(60.67,122.11) | 2.30(1.88,2.84) | 0.04(0.03,0.05) | -13.78(-14.43,-13.11) |
|  | Male | 4059.41(3205.65,5214.06) | 58.00(36.43,87.28) | 2.19(1.72,2.78) | 0.03(0.02,0.04) | -13.31(-14.02,-12.60) |
|  | Female | 3289.52(2590.82,4181.57) | 29.01(21.53,38.21) | 2.40(1.90,3.09) | 0.05(0.03,0.07) | 14.46(-15.06,-13.85) |
| Enteric infections | Both | 77185.80(56467.00,98479.93) | 1341.99(951.79,1906.44) | 24.17(17.68,30.85) | 0.57(0.41,0.80) | -12.33(-12.76,-11.91) |
|  | Male | 40223.45(24923.65,56617.01) | 716.15(457.11,1102.90) | 23.79(14.74,33.49) | 0.57(0.36,0.87) | -12.09(-12.42,-11.76) |
|  | Female | 36962.35(28125.27,46852.42) | 625.84(435.47,880.96) | 24.61(18.73,31.19) | 0.58(0.40,0.80) | -12.61(-13.18,-12.03) |
| HIV/AIDS | Both | 62.06(17.87,108.58) | 554.73(273.91,1108.13) | 0.02(0.01,0.03) | 0.24(0.12,0.47) | 8.28(6.94,9.62) |
|  | Male | 32.83(9.46,57.42) | 294.47(145.54,588.99) | 0.02(0.01,0.03) | 0.23(0.11,0.47) | 8.24(6.92,9.59) |
|  | Female | 29.23(8.37,51.19) | 260.25(128.41,518.71) | 0.02(0.01,0.03) | 0.24(0.12,0.48) | 8.31(6.97,9.66) |
| Lower respiratory infections | Both | 326569.43(278247.95,381652.68) | 11668.04(9317.97,14362.36) | 102.28(87.15,119.54) | 5.11(4.07,6.29) | -10.04(-10.41,-9.67) |
|  | Male | 179862.45(150378.92,210379.62) | 7053.83(5558.68,8715.38) | 106.38(88.94,124.43) | 5.77(4.54,7.13) | -9.77(-10.12,-9.42) |
|  | Female | 146706.98(124438.84,172676.90) | 4614.21(3588.44,5796.70) | 97.69(82.87,114.98) | 4.35(3.38,5.46) | -10.41(-10.80,-10.02) |
| Neglected tropical diseases and malaria | Both | 2562.37(1607.78,5587.09) | 225.75(104.68,484.85) | 0.81(0.51,1.76) | 0.09(0.04,0.19) | -6.24(-7.15,-5.31) |
|  | Male | 1494.85(865.15,3530.45) | 147.10(61.57,333.64) | 0.89(0.52,2.13) | 0.11(0.05,0.25) | -5.84(-6.76,-4.91) |
|  | Female | 1067.52(699.48,2212.46) | 78.66(39.39,159.26) | 0.71(0.47,1.46) | 0.07(0.03,0.14) | -6.87(-7.81,-5.93) |
| Other infectious diseases | Both | 82274.77(54086.67,121594.19) | 4412.81(3161.23,6425.35) | 25.81(16.96,38.16) | 1.88(1.34,2.75) | -8.67(-8.89,-8.44) |
|  | Male | 46082.64(29768.29,66484.53) | 2540.24(1774.11,3708.74) | 27.34(17.66,39.46) | 2.03(1.41,2.97) | -8.63(-8.87,-8.39) |
|  | Female | 36192.13(23210.97,57485.58) | 1872.57(1287.80,2793.22) | 24.09(15.45,38.27) | 1.72(1.17,2.57) | -8.73(-8.94,-8.51) |
| Sexually transmitted infections excluding HIV | Both | 4267.40(1514.10,8963.67) | 1866.76(673.49,4139.42) | 1.34(0.47,2.81) | 0.84(0.30,1.86) | -2.23(-2.88,-1.58) |
|  | Male | 2303.05(815.22,4902.63) | 1019.25(355.27,2222.27) | 1.36(0.48,2.90) | 0.86(0.30,1.87) | -2.19(-2.83,-1.56) |
|  | Female | 1964.35(690.86,4054.39) | 847.51(311.05,1918.90) | 1.31(0.46,2.70) | 0.82(0.30,1.86) | -2.28(-2.94,-1.62) |
| Tuberculosis | Both | 20699.44(17280.40,24398.68) | 310.80(248.59,393.09) | 6.49(5.41,7.65) | 0.13(0.10,0.17) | -12.18(-12.61,-11.74) |
|  | Male | 10265.86(7808.98,13229.10) | 188.26(140.67,258.16) | 6.09(4.63,7.85) | 0.15(0.11,0.20) | -11.55(-11.97,-11.12) |
|  | Female | 10433.58(8581.70,12515.77) | 122.55(93.49,153.45) | 6.93(5.70,8.31) | 0.11(0.08,0.14) | -12.96(-13.43,-12.49) |
| Upper respiratory infections | Both | 7228.89(1572.85,10989.30) | 146.93(88.30,311.49) | 2.27(0.49,3.44) | 0.06(0.04,0.14) | -12.53(-13.19,-11.87) |
|  | Male | 3834.33(668.20,6034.48) | 93.75(52.28,216.29) | 2.27(0.40,3.57) | 0.08(0.04,0.18) | -11.95(-12.57,-11.32) |
|  | Female | 3394.56(630.43,5210.98) | 53.18(29.85,130.65) | 2.26(0.42,3.47) | 0.05(0.03,0.12) | -13.35(-14.07,-12.62) |
| Otitis media | Both | 10.06(2.98,20.33) | 0.12(0.03,0.27) | 0.00(0.00,0.01) | 0.00(0.00,0.00) | -11.91(-14.55,-9.18) |
|  | Male | 3.84(0.19,9.39) | 0.01(0.00,0.06) | 0.00(0.00,0.01) | 0.00(0.00,0.00) | -16.79(-18.56,-14.98) |
|  | Female | 6.22(2.08,11.95) | 0.10(0.03,0.23) | 0.00(0.00,0.01) | 0.00(0.00,0.00) | -10.34(-13.26,-7.32) |

Table 3: Burden of DALYs caused by infectious diseases among children in China

|  |  | DALYs(95% UI) |  |  |  |  |  |
| --- | --- | --- | --- | --- | --- | --- | --- |
|  |  | Cases in 1990 (million) | Cases in 2021(million) | ASR in 1990 (per 100 000) | ASR in 2021 (per 100 000) | EAPC (95% CI) |  |
|  |  |  |  |  |  |  |  |
| Acute hepatitis | Both | 675327.46(551558.41,830390.47) | 22200.62(15196.14,31886.54) | 211.59(172.82,260.15) | 8.69(5.98,12.42) | -11.00(-11.77,-10.22) |  |
|  | Male | 372353.23(294752.74,476242.67) | 12882.12(8399.14,19197.34) | 220.59(174.64,282.10) | 9.53(6.22,14.16) | -10.84(-11.62,-10.05) |  |
|  | Female | 302974.23(239471.56,384208.26) | 9318.50(6286.88,13528.50) | 201.50(159.27,255.53) | 7.72(5.25,11.11) | -11.22(-11.98,-10.45) |  |
| Enteric infections | Both | 7205894.23(5383549.56,9135123.03) | 161785.99(118441.94,215695.79) | 2257.18(1686.09,2861.65) | 67.34(49.64,89.26) | -11.71(-12.16,-11.27) |  |
|  | Male | 3761989.42(2386885.21,5220773.22) | 90066.82(61779.05,126926.46) | 2226.17(1412.23,3089.89) | 69.68(47.91,98.06) | -11.38(-11.73,-11.03) |  |
|  | Female | 3443904.81(2644686.57,4323423.96) | 71719.16(52303.83,96014.25) | 2292.41(1760.58,2877.59) | 64.69(47.51,86.09) | -12.10(-12.68,-11.52) |  |
| HIV/AIDS | Both | 5486.13(1579.24,9571.47) | 48277.97(23809.13,96537.31) | 1.72(0.49,3.00) | 20.55(10.04,41.38) | 8.23(6.90,9.58) |  |
|  | Male | 2902.42(835.97,5065.00) | 25627.92(12650.60,51259.10) | 1.72(0.49,3.00) | 20.39(9.97,41.06) | 8.20(6.87,9.54) |  |
|  | Female | 2583.71(739.73,4512.42) | 22650.05(11161.88,45205.96) | 1.72(0.49,3.01) | 20.74(10.12,41.69) | 8.26(6.93,9.62) |  |
| Lower respiratory infections | Both | 29052423.19(24778410.76,33923584.15) | 1039781.93(830852.74,1278317.88) | 9099.27(7760.29,10624.83) | 455.96(363.74,560.93) | -10.03(-10.39,-9.66) |  |
|  | Male | 16004988.98(13383790.19,18714063.85) | 627731.96(496448.63,774258.97) | 9465.27(7915.35,11067.24) | 513.91(405.99,634.10) | -9.76(-10.11,-9.41) |  |
|  | Female | 13047434.21(11081586.19,15352379.61) | 412049.97(321415.21,516112.92) | 8688.41(7380.01,10223.23) | 389.11(303.13,486.96) | -10.39(-10.78,-10.01) |  |
| Neglected tropical diseases and malaria | Both | 778759.21(500783.68,1161610.05) | 79779.92(52764.83,115412.08) | 244.70(157.37,365.16) | 30.81(20.37,44.55) | -6.75(-6.98,-6.52) |  |
|  | Male | 448129.00(290391.17,681422.73) | 43739.08(28154.76,66633.82) | 269.44(174.45,410.23) | 31.70(20.39,48.29) | -6.89(-7.12,-6.67) |  |
|  | Female | 330630.22(213263.23,487015.86) | 36040.84(24272.09,52142.18) | 217.45(140.47,320.11) | 29.79(20.08,43.09) | -6.57(-6.83,-6.32) |  |
| Other infectious diseases | Both | 7429089.97(4960868.96,10856690.82) | 439477.68(323356.46,619343.48) | 2475.31(1624.12,3538.86) | 197.70(141.66,283.19) | -8.39(-8.61,-8.17) |  |
|  | Male | 4172191.71(2736515.69,5962369.50) | 249966.24(180465.73,355962.94) | 2167.50(1402.14,3411.41) | 171.63(121.53,249.17) | -8.39(-8.63,-8.15) |  |
|  | Female | 3256898.25(2107556.47,5124757.19) | 189511.44(135217.26,273303.93) | 2330.22(1555.90,3406.48) | 185.59(135.67,263.22) | -8.39(-8.60,-8.18) |  |
| Sexually transmitted infections excluding HIV | Both | 384506.27(136790.97,807016.62) | 168324.76(60846.69,372948.73) | 120.36(42.82,252.63) | 75.72(27.36,167.80) | -2.23(-2.87,-1.58) |  |
|  | Male | 207655.00(73687.26,441753.55) | 92020.82(32130.09,200498.93) | 122.66(43.53,260.93) | 77.22(26.95,168.26) | -2.19(-2.82,-1.55) |  |
|  | Female | 176851.27(62377.47,364719.43) | 76303.94(28044.21,172651.74) | 117.78(41.54,242.91) | 74.00(27.19,167.46) | -2.28(-2.94,-1.62) |  |
| Tuberculosis | Both | 1894461.56(1591156.72,2233264.29) | 39682.00(31939.40,49459.33) | 593.77(498.70,699.96) | 16.69(13.46,20.78) | -11.36(-11.69,-11.02) |  |
|  | Male | 938294.29(721505.10,1201357.90) | 22882.19(17923.23,29427.76) | 556.85(428.18,712.81) | 18.02(14.14,23.14) | -10.85(-11.18,-10.51) |  |
|  | Female | 956167.27(786247.63,1144074.70) | 16799.81(12922.43,20976.18) | 635.18(522.28,760.07) | 15.17(11.68,18.87) | -11.96(-12.32,-11.60) |  |
| Upper respiratory infections | Both | 869859.18(344911.27,1280092.53) | 198134.54(113578.72,323029.00) | 272.88(108.36,401.58) | 78.59(45.38,127.69) | -4.61(-5.00,-4.22) |  |
|  | Male | 462065.65(169803.36,690257.06) | 108476.96(62467.41,175934.36) | 274.67(101.39,410.37) | 80.66(46.78,130.27) | -4.54(-4.92,-4.16) |  |
|  | Female | 407793.53(143150.23,601659.72) | 89657.58(51147.75,148113.89) | 271.00(95.02,399.72) | 76.20(43.80,125.56) | -4.69(-5.10,-4.28) |  |
| Otitis media | Both | 148522.48(84423.81,238569.88) | 109165.03(61511.57,175859.91) | 46.71(26.55,75.04) | 41.96(23.65,67.72) | -0.41(-0.46,-0.36) |  |
|  | Male | 82464.21(47306.99,132984.34) | 62221.44(35600.68,100966.83) | 9.69(28.51,80.13) | 44.69(25.56,72.59) | -0.40(-0.45,-0.35) |  |
|  | Female | 66058.27(37323.25,106955.91) | 46943.59(25904.16,76247.93) | 43.50(24.57,70.46) | 38.84(21.44,63.20) | -0.44(-0.49,-0.39) |  |

Table 4: Age-specific burden of infectious diseases incidence rate among Chinese children

| Age groups | | | | | | | | | | | | | |
| --- | --- | --- | --- | --- | --- | --- | --- | --- | --- | --- | --- | --- | --- |
|  |  | ASIR | | | | | | Number of incidence | | | | | |
|  |  | <5 | | 5-9 | | 10-14 | | <5 | | 5-9 | | 10-14 | |
|  |  | 1990 | 2021 | 1990 | 2021 | 1990 | 2021 | 1990 | 2021 | 1990 | 2021 | 1990 | 2021 |
| Acute hepatitis | Male | 14027.92(12521.84,15503.38) | 7589.33(6647.31,8553.85) | 7532.41(6788.62,8409.18) | 4765.79(4177.87,5312.72) | 6075.76(5192.29,7205.93) | 3422.16(3043.62,3861.82) | 8313345.76(7420803.39,9187747.65) | 3159487.46(2767317.22,3561022.06) | 4085529.08(3682105.85,4561083.24) | 2427834.93(2128329.73,2706455.60) | 3207960.00(2741493.43,3804679.44) | 1573939.21(1399836.97,1776146.60) |
|  | Female | 13666.49(12294.32,15087.38) | 7641.30(6689.07,8598.11) | 7098.67(6410.10,7948.58) | 4735.79(4169.07,5268.61) | 5385.60(4650.92,6390.70) | 3354.27(2962.31,3772.75) | 7180806.75(6459825.03,7927385.01) | 2753741.81(2410582.76,3098556.74) | 3552070.91(3207523.05,3977358.36) | 2122932.96(1868886.98,2361783.98) | 2665501.52(2301884.17,3162959.52) | 1348433.53(1190862.46,1516661.57) |
|  | Both | 13858.07(12434.15,15267.48) | 7613.44(6694.23,8594.84) | 7324.27(6639.16,8028.49) | 4751.75(4175.04,5291.96) | 5741.83(4991.58,6570.00) | 3390.50(3004.29,3819.34) | 15494152.52(13902130.90,17069959.70) | 5913229.27(5199289.92,6675461.51) | 7637599.99(6923178.86,8371947.71) | 4550767.88(3998450.72,5068129.54) | 5873461.51(5106012.01,6720608.76) | 2922372.74(2589490.37,3292004.47) |
| Enteric infections | Male | 107027.38(84212.34,134572.51) | 13843.05(9906.67,18907.54) | 44968.98(28888.05,63751.67) | 12102.24(7248.47,17428.20) | 29942.74(18618.94,46507.57) | 8633.75(5105.03,13257.80) | 63427494.35(49906647.59,79751526.07) | 5762950.65(4124210.01,7871332.21) | 24390885.53(15668695.03,34578499.09) | 6165234.23(3692581.03,8878436.10) | 15809553.82(9830664.99,24555665.48) | 3970876.79(2347933.01,6097595.81) |
|  | Female | 102174.84(79762.38,128802.86) | 9737.02(6931.67,13194.13) | 42467.55(27048.10,60022.41) | 8202.79(4856.02,11941.17) | 30614.95(19179.61,48100.38) | 6320.49(3805.31,9692.96) | 53685890.22(41909672.60,67677092.73) | 3508991.42(2498008.45,4754850.81) | 21250157.37(13534486.36,30034360.30) | 3677101.65(2176831.61,5352922.44) | 15152295.81(9492592.50,23806387.39) | 2540864.00(1529751.64,3896613.35) |
|  | Both | 104746.94(82452.17,132201.76) | 11937.87(8545.57,16273.74) | 43768.65(28087.73,62129.80) | 10277.01(6133.65,14885.86) | 30267.98(18907.89,47237.02) | 7554.84(4498.97,11568.68) | 117113384.56(92186491.17,147809525.81) | 9271942.07(6637197.90,12639537.34) | 45641042.91(29289307.67,64787674.35) | 9842335.88(5874224.78,14256246.17) | 30961849.63(19341336.85,48319885.08) | 6511740.79(3877792.93,9971389.13) |
| HIV/AIDS | Male | 0.20(0.10,0.32) | 1.33(0.61,2.79) | 0.00(0.00,0.00) | 0.00(0.00,0.00) | 0.00(0.00,0.00) | 0.00(0.00,0.00) | 119.48(58.00,192.52) | 553.28(255.52,1161.24) | 0.00(0.00,0.00) | 0.00(0.00,0.00) | 0.00(0.00,0.00) | 0.00(0.00,0.00) |
|  | Female | 0.19(0.09,0.31) | 1.26(0.59,2.64) | 0.00(0.00,0.00) | 0.00(0.00,0.00) | 0.00(0.00,0.00) | 0.00(0.00,0.00) | 100.75(48.76,163.36) | 454.80(211.71,949.95) | 0.00(0.00,0.00) | 0.00(0.00,0.00) | 0.00(0.00,0.00) | 0.00(0.00,0.00) |
|  | Both | 0.20(0.10,0.32) | 1.30(0.60,2.72) | 0.00(0.00,0.00) | 0.00(0.00,0.00) | 0.00(0.00,0.00) | 0.00(0.00,0.00) | 220.23(106.76,356.00) | 1008.08(466.07,2116.42) | 0.00(0.00,0.00) | 0.00(0.00,0.00) | 0.00(0.00,0.00) | 0.00(0.00,0.00) |
| Lower respiratory infections | Male | 15623.14(13786.65,17702.75) | 2888.60(2412.16,3390.66) | 5082.66(4011.21,6449.41) | 2826.40(2153.62,3703.57) | 1944.44(1504.51,2488.92) | 1447.05(1060.04,2007.80) | 9258722.44(8170362.39,10491155.23) | 1202541.72(1004198.74,1411551.92) | 2756801.44(2175654.63,3498116.48) | 1439853.40(1097118.83,1886708.57) | 1026649.15(794370.42,1314133.81) | 665534.87(487537.73,923435.85) |
|  | Female | 17783.39(15727.35,20311.45) | 3319.98(2753.85,3935.67) | 4922.37(3829.41,6318.59) | 2938.93(2209.18,3856.60) | 1914.94(1458.56,2546.19) | 1563.01(1136.24,2164.76) | 9343952.81(8263644.50,10672277.53) | 1196443.47(992420.80,1418322.80) | 2463084.96(1916184.50,3161731.61) | 1317446.37(990317.37,1728813.50) | 947764.10(721887.66,1260191.63) | 628338.83(456775.44,870242.30) |
|  | Both | 16638.35(14661.88,18916.42) | 3088.76(2571.93,3649.69) | 5005.74(3938.13,6409.70) | 2879.07(2166.32,3766.99) | 1930.17(1477.70,2511.13) | 1501.14(1097.18,2078.52) | 18602675.25(16392870.15,21149701.05) | 2398985.19(1997575.87,2834649.53) | 5219886.40(4106597.32,6683905.74) | 2757299.77(2074692.93,3607662.37) | 1974413.25(1511574.43,2568692.73) | 1293873.70(945688.87,1791535.14) |
| Neglected tropical diseases and malaria | Male | 11.69(9.58,18.37) | 1.88(0.72,5.43) | 15.95(13.23,23.35) | 3.29(1.34,9.06) | 22.77(18.87,29.79) | 3.61(1.58,9.16) | 6928.11(5677.81,10885.77) | 782.72(298.95,2262.61) | 8649.80(7175.08,12665.43) | 1676.62(682.96,4613.07) | 12022.65(9962.24,15727.36) | 1660.96(728.33,4211.02) |
|  | Female | 10.82(8.76,17.48) | 1.93(0.64,6.09) | 14.96(12.31,21.80) | 3.38(1.14,10.21) | 22.18(18.24,29.27) | 3.59(1.36,9.93) | 5686.48(4603.16,9184.88) | 695.45(230.27,2194.05) | 7485.01(6157.50,10908.89) | 1516.58(511.47,4576.25) | 10976.87(9029.38,14488.02) | 1444.65(544.81,3991.24) |
|  | Both | 11.28(9.25,17.88) | 1.90(0.77,5.11) | 15.47(12.81,22.61) | 3.33(1.43,8.56) | 22.48(18.57,29.59) | 3.60(1.62,8.43) | 12614.59(10338.75,19995.45) | 1478.17(596.70,3971.86) | 16134.81(13355.79,23574.05) | 3193.20(1369.50,8202.19) | 22999.52(18991.73,30263.56) | 3105.61(1398.06,7264.50) |
| Other infectious diseases | Male | 25393.22(23317.30,27266.21) | 15815.47(14425.73,17210.41) | 8766.27(7829.94,9771.91) | 5760.88(4991.28,6635.19) | 6432.19(5485.19,7591.28) | 3704.60(3304.59,4181.32) | 15048749.60(13818500.78,16158737.86) | 6584081.93(6005524.08,7164807.89) | 4754770.80(4246909.60,5300220.31) | 2934759.86(2542702.90,3380160.81) | 3396150.12(2896141.39,4008141.22) | 1703837.68(1519861.86,1923093.87) |
|  | Female | 25518.75(23563.94,27201.76) | 16025.48(14603.04,17304.28) | 8313.70(7494.85,9281.07) | 5611.57(4886.16,6424.56) | 5720.40(4938.65,6834.24) | 3601.84(3193.80,4041.00) | 13408355.69(12381238.13,14292666.51) | 5775201.92(5262589.95,6236052.43) | 4160055.06(3750317.02,4644115.02) | 2515525.41(2190339.45,2879966.65) | 2831207.40(2444291.61,3382481.99) | 1447956.70(1283923.71,1624500.39) |
|  | Both | 25452.21(23465.51,27222.16) | 15912.91(14504.33,17264.34) | 8549.10(7715.10,9529.41) | 5690.99(4955.36,6530.03) | 6087.80(5313.42,6954.73) | 3656.67(3257.98,4121.66) | 28457105.28(26235853.97,30436013.32) | 12359283.85(11265265.60,13408917.75) | 8914825.85(8045149.91,9937069.09) | 5450285.27(4745763.79,6253831.12) | 6227357.53(5435224.99,7114160.42) | 3151794.38(2808148.18,3552576.59) |
| Otitis media | Male | 16661.32(10870.15,24920.68) | 16292.47(10088.12,25078.26) | 8564.59(4277.49,14806.96) | 8564.28(4277.81,14806.43) | 4121.28(1947.42,7556.82) | 4121.09(1946.28,7555.34) | 9873975.07(6441964.22,14768709.91) | 6782662.92(4199748.96,10440244.31) | 4645379.22(2320083.96,8031200.81) | 4362894.02(2179243.43,7542830.09) | 2176006.95(1028221.25,3989946.11) | 1895393.96(895141.17,3474892.09) |
|  | Female | 18987.98(11809.07,28817.41) | 18599.59(11155.67,28431.13) | 9779.54(5023.56,17339.42) | 9779.12(5023.35,17338.79) | 4710.84(2311.00,8506.19) | 4710.91(2310.99,8505.55) | 9976885.03(6204858.09,15141576.77) | 6702852.31(4020240.54,10245904.37) | 4893543.00(2513715.42,8676398.30) | 4383731.17(2251838.18,7772539.56) | 2331540.86(1143787.64,4209981.62) | 1893807.69(929027.32,3419268.88) |
|  | Both | 17754.73(11351.08,26286.39) | 17362.96(10703.43,26164.97) | 9147.59(4658.56,15976.22) | 9132.91(4648.98,15945.31) | 4406.53(2159.53,8095.09) | 4396.18(2150.87,8071.38) | 19850860.09(12691188.88,29389769.10) | 13485515.23(8313170.49,20321883.78) | 9538922.22(4857853.11,16659676.61) | 8746625.19(4452343.16,15270881.73) | 4507547.81(2209035.26,8280661.37) | 3789201.65(1853900.46,6956960.03) |
| Sexually transmitted infections excluding HIV | Male | 20.04(12.90,28.66) | 15.22(10.82,20.52) | 0.19(0.03,0.57) | 0.19(0.03,0.58) | 770.77(432.92,1318.45) | 715.28(402.76,1229.24) | 11875.21(7643.91,16986.21) | 6334.11(4503.45,8542.33) | 104.10(17.22,310.82) | 98.45(16.64,295.52) | 406958.86(228580.98,696132.83) | 328972.99(185237.79,565359.37) |
|  | Female | 19.26(12.32,27.61) | 14.84(10.56,19.97) | 0.17(0.03,0.52) | 0.17(0.03,0.52) | 743.02(448.09,1191.79) | 707.47(434.65,1105.46) | 10118.82(6475.74,14508.42) | 5347.59(3805.61,7195.21) | 83.16(13.69,259.50) | 75.15(12.09,235.08) | 367743.37(221775.34,589853.24) | 284406.19(174732.80,444398.76) |
|  | Both | 19.67(12.67,28.14) | 15.04(10.70,20.23) | 0.18(0.03,0.54) | 0.18(0.03,0.56) | 757.34(442.30,1248.38) | 711.63(420.98,1178.48) | 21994.03(14163.64,31457.37) | 11681.70(8309.77,15714.01) | 187.26(33.52,567.04) | 173.60(31.35,533.29) | 774702.22(452443.52,1276994.34) | 613379.18(362855.36,1015764.84) |
| Tuberculosis | Male | 68.41(51.88,87.65) | 11.86(9.35,14.93) | 31.42(19.94,46.62) | 5.03(3.28,7.43) | 51.07(32.12,74.59) | 9.44(5.78,14.21) | 40541.88(30743.58,51941.23) | 4936.16(3892.73,6213.79) | 17042.73(10816.17,25284.78) | 2564.37(1669.39,3784.78) | 26964.78(16956.99,39381.21) | 4339.68(2659.59,6533.60) |
|  | Female | 72.61(54.36,93.91) | 13.26(10.44,16.78) | 36.73(23.03,54.30) | 6.09(4.02,9.11) | 58.40(35.83,86.94) | 11.33(7.01,17.02) | 38149.79(28561.55,49342.08) | 4778.23(3760.82,6046.43) | 18377.95(11525.37,27171.91) | 2731.27(1803.77,4082.52) | 28902.91(17732.01,43030.13) | 4555.90(2818.26,6843.59) |
|  | Both | 70.38(52.60,90.42) | 12.51(9.87,15.77) | 33.97(21.20,50.33) | 5.53(3.63,8.18) | 54.62(33.85,80.12) | 10.32(6.37,15.56) | 78691.67(58808.61,101094.60) | 9714.39(7664.45,12247.09) | 35420.69(22103.07,52486.85) | 5295.64(3479.85,7836.96) | 55867.70(34627.88,81961.19) | 8895.58(5488.12,13412.70) |
| Upper respiratory infections | Male | 279514.35(218558.22,344821.54) | 272733.97(207912.66,339350.34) | 200261.00(136916.49,272532.85) | 197713.98(134610.62,267855.72) | 147450.24(103004.33,202614.24) | 145677.22(101590.89,201730.10) | 165648212.42(129523860.23,204351124.12) | 113540933.92(86555399.04,141273765.98) | 108620291.46(74262631.69,147820081.73) | 100721305.00(68574601.81,136453560.96) | 77852668.53(54385546.06,106978864.85) | 67000602.39(46724197.88,92780727.09) |
|  | Female | 264775.24(205243.93,326330.82) | 264007.48(201230.29,331279.34) | 193925.27(132097.50,261558.24) | 192970.65(131696.02,261686.87) | 142907.07(98544.83,198302.18) | 141844.41(98730.46,197299.73) | 139121276.88(107841646.44,171464523.45) | 95142041.09(72518627.34,119385223.16) | 97037445.16(66099710.59,130880019.05) | 86503833.84(59035977.46,117307568.10) | 70729187.14(48772922.75,98145961.76) | 57022088.40(39690086.75,79315377.57) |
|  | Both | 272587.72(212517.44,336024.32) | 268684.93(205371.60,335103.63) | 197220.75(134370.27,267207.40) | 195493.76(133222.98,264968.25) | 145252.07(100846.64,200420.17) | 143889.59(100613.43,199607.29) | 304769489.30(237607295.48,375695427.81) | 208682975.01(159508596.28,260269241.97) | 205657736.62(140118552.76,278638367.33) | 187225138.84(127588166.41,253761129.06) | 148581855.68(103158468.81,205014640.90) | 124022690.79(86721692.07,172047420.87) |

Table 5: Age-specific burden of infectious disease mortality among children in China

| Age groups | | | | | | | | | | | | | |
| --- | --- | --- | --- | --- | --- | --- | --- | --- | --- | --- | --- | --- | --- |
|  |  | ASMR | | | | | | Number of Dealth | | | | | |
|  |  | <5 | | 5-9 | | 10-14 | | <5 | | 5-9 | | 10-14 | |
|  |  | 1990 | 2021 | 1990 | 2021 | 1990 | 2021 | 1990 | 2021 | 1990 | 2021 | 1990 | 2021 |
| Acute hepatitis | Male | 6.31(4.98,8.11) | 0.12(0.07,0.18) | 0.38(0.30,0.47) | 0.01(0.01,0.02) | 0.22(0.17,0.29) | 0.01(0.01,0.01) | 3737.95(2953.33,4806.14) | 48.86(30.10,74.09) | 204.91(164.43,255.52) | 5.49(3.73,8.02) | 116.54(87.89,152.41) | 3.66(2.60,5.17) |
|  | Female | 5.79(4.56,7.37) | 0.06(0.05,0.09) | 0.30(0.24,0.38) | 0.01(0.01,0.01) | 0.19(0.15,0.25) | 0.01(0.00,0.01) | 3043.27(2397.20,3871.15) | 23.34(17.11,30.85) | 150.97(120.40,188.78) | 3.18(2.53,4.04) | 95.28(73.22,121.64) | 2.49(1.89,3.31) |
|  | Both | 6.07(4.94,7.49) | 0.09(0.06,0.13) | 0.34(0.28,0.41) | 0.01(0.01,0.01) | 0.21(0.17,0.25) | 0.01(0.01,0.01) | 6781.22(5518.25,8378.76) | 72.20(49.35,102.67) | 355.88(294.05,424.12) | 8.67(6.48,11.54) | 211.83(174.88,256.13) | 6.15(4.84,7.90) |
| Enteric infections | Male | 64.84(40.63,90.77) | 1.22(0.80,1.84) | 2.22(1.02,3.52) | 0.26(0.15,0.41) | 1.12(0.56,1.73) | 0.16(0.10,0.27) | 38427.72(24075.91,53792.05) | 509.78(334.30,767.16) | 1202.67(551.97,1910.72) | 131.17(78.10,209.38) | 593.06(295.78,914.24) | 75.20(44.70,126.36) |
|  | Female | 67.52(52.33,84.52) | 1.31(0.96,1.73) | 1.97(0.81,3.30) | 0.22(0.12,0.36) | 1.01(0.45,1.60) | 0.14(0.08,0.24) | 35476.18(27494.07,44410.78) | 472.33(346.16,624.40) | 986.94(407.23,1649.50) | 96.67(55.80,161.99) | 499.23(223.97,792.15) | 56.83(33.51,94.56) |
|  | Both | 66.10(48.98,83.75) | 1.26(0.94,1.70) | 2.10(1.04,3.15) | 0.24(0.14,0.38) | 1.07(0.60,1.53) | 0.15(0.09,0.26) | 73903.90(54761.13,93631.98) | 982.11(732.94,1322.13) | 2189.61(1088.02,3282.48) | 227.84(137.51,363.70) | 1092.29(617.86,1565.47) | 132.04(81.34,220.61) |
| HIV/AIDS | Male | 0.05(0.02,0.09) | 0.52(0.24,1.08) | 0.00(0.00,0.01) | 0.09(0.05,0.17) | 0.00(0.00,0.00) | 0.07(0.04,0.12) | 31.04(9.46,53.09) | 215.10(99.87,449.79) | 1.77(0.00,4.28) | 47.73(26.79,86.19) | 0.02(0.00,0.05) | 31.64(18.88,53.01) |
|  | Female | 0.05(0.02,0.09) | 0.53(0.24,1.10) | 0.00(0.00,0.01) | 0.10(0.05,0.17) | 0.00(0.00,0.00) | 0.07(0.04,0.12) | 27.60(8.37,47.22) | 189.38(87.61,394.74) | 1.61(0.00,3.93) | 42.70(24.01,76.94) | 0.02(0.00,0.05) | 28.18(16.79,47.04) |
|  | Both | 0.05(0.02,0.09) | 0.52(0.24,1.09) | 0.00(0.00,0.01) | 0.09(0.05,0.17) | 0.00(0.00,0.00) | 0.07(0.04,0.12) | 58.64(17.87,100.26) | 404.48(187.48,844.95) | 3.38(0.00,8.22) | 90.42(50.79,163.13) | 0.04(0.00,0.10) | 59.82(35.64,100.05) |
| Lower respiratory infections | Male | 291.03(243.18,340.48) | 14.68(11.52,18.16) | 9.76(8.34,11.32) | 1.23(0.99,1.51) | 3.97(3.30,4.66) | 0.69(0.56,0.83) | 172474.45(144115.21,201775.30) | 6110.73(4796.84,7561.42) | 5292.93(4523.90,6142.43) | 625.13(503.14,770.54) | 2095.07(1739.81,2461.90) | 317.97(258.70,383.42) |
|  | Female | 269.14(230.83,316.48) | 11.11(8.60,13.87) | 7.49(4.59,9.03) | 0.91(0.73,1.19) | 3.13(1.73,3.78) | 0.50(0.40,0.66) | 141414.31(121286.44,166290.20) | 4005.54(3100.61,4998.04) | 3745.46(2297.12,4516.06) | 406.67(327.45,531.86) | 1547.21(855.28,1870.64) | 202.00(160.38,266.80) |
|  | Both | 280.74(239.94,328.28) | 13.02(10.32,16.09) | 8.67(6.82,10.01) | 1.08(0.90,1.30) | 3.56(2.81,4.09) | 0.60(0.51,0.73) | 313888.76(268268.62,367033.40) | 10116.26(8015.55,12493.84) | 9038.39(7107.92,10433.70) | 1031.80(863.71,1242.30) | 3642.28(2871.41,4185.57) | 519.97(438.71,626.22) |
| Neglected tropical diseases and malaria | Male | 1.61(0.99,2.95) | 0.16(0.07,0.34) | 0.71(0.36,2.43) | 0.10(0.04,0.24) | 0.30(0.15,0.88) | 0.07(0.03,0.15) | 954.73(587.90,1745.91) | 67.23(27.24,142.23) | 383.43(197.73,1318.56) | 49.64(21.04,121.36) | 156.69(79.53,465.98) | 30.23(13.29,70.06) |
|  | Female | 1.44(1.02,2.22) | 0.12(0.06,0.22) | 0.41(0.23,1.14) | 0.05(0.03,0.12) | 0.21(0.10,0.97) | 0.03(0.02,0.07) | 758.80(537.50,1164.97) | 41.97(19.85,79.47) | 203.48(114.80,569.81) | 24.33(13.02,53.44) | 105.24(47.18,477.68) | 12.36(6.52,26.35) |
|  | Both | 1.53(1.02,2.55) | 0.14(0.06,0.28) | 0.56(0.31,1.87) | 0.08(0.04,0.18) | 0.26(0.13,0.76) | 0.05(0.02,0.11) | 1713.53(1144.26,2855.61) | 109.20(47.87,218.82) | 586.92(326.93,1950.44) | 73.96(35.85,173.51) | 261.93(136.59,781.05) | 42.58(20.95,92.52) |
| Other infectious diseases | Male | 68.42(43.71,97.46) | 4.61(3.13,6.88) | 7.38(5.01,11.96) | 0.84(0.63,1.16) | 2.90(2.17,4.24) | 0.42(0.33,0.55) | 40548.89(25905.31,57759.66) | 1920.75(1303.80,2864.74) | 4001.08(2718.38,6484.34) | 426.13(320.16,590.62) | 1532.66(1144.60,2240.53) | 193.37(150.15,253.37) |
|  | Female | 60.19(38.67,94.66) | 3.88(2.57,5.88) | 6.79(4.12,11.93) | 0.74(0.55,1.08) | 2.36(1.68,3.60) | 0.35(0.28,0.47) | 31627.73(20316.12,49735.22) | 1396.95(927.41,2118.80) | 3397.88(2064.01,5967.45) | 333.26(247.62,486.32) | 1166.52(830.85,1782.90) | 142.37(112.77,188.10) |
|  | Both | 64.56(42.27,93.95) | 4.27(2.96,6.38) | 7.10(4.62,12.02) | 0.79(0.61,1.09) | 2.64(1.97,3.93) | 0.39(0.32,0.49) | 72176.63(47258.34,105042.35) | 3317.70(2297.65,4954.01) | 7398.97(4813.89,12533.27) | 759.38(587.14,1048.19) | 2699.18(2014.44,4018.57) | 335.73(276.45,423.15) |
| Otitis media | Male | 0.01(0.00,0.01) | 0.00(0.00,0.00) | 0.00(0.00,0.00) | 0.00(0.00,0.00) | 0.00(0.00,0.00) | 0.00(0.00,0.00) | 3.58(0.17,8.78) | 0.01(0.00,0.04) | 0.13(0.01,0.30) | 0.00(0.00,0.01) | 0.13(0.01,0.31) | 0.00(0.00,0.01) |
|  | Female | 0.01(0.00,0.01) | 0.00(0.00,0.00) | 0.00(0.00,0.01) | 0.00(0.00,0.00) | 0.00(0.00,0.01) | 0.00(0.00,0.00) | 3.50(1.35,6.19) | 0.03(0.01,0.08) | 1.25(0.38,2.55) | 0.03(0.01,0.07) | 1.47(0.35,3.22) | 0.04(0.01,0.09) |
|  | Both | 0.01(0.00,0.01) | 0.00(0.00,0.00) | 0.00(0.00,0.00) | 0.00(0.00,0.00) | 0.00(0.00,0.00) | 0.00(0.00,0.00) | 7.08(2.11,14.11) | 0.05(0.01,0.10) | 1.38(0.44,2.72) | 0.03(0.01,0.07) | 1.60(0.44,3.50) | 0.04(0.01,0.09) |
| Sexually transmitted infections excluding HIV | Male | 3.87(1.37,8.23) | 2.44(0.85,5.31) | 0.02(0.01,0.04) | 0.01(0.00,0.02) | 0.00(0.00,0.00) | 0.00(0.00,0.00) | 2291.59(810.88,4878.79) | 1014.88(353.72,2212.37) | 10.34(3.66,22.18) | 4.24(1.48,9.69) | 1.12(0.69,1.67) | 0.12(0.06,0.22) |
|  | Female | 3.72(1.31,7.67) | 2.34(0.86,5.30) | 0.02(0.01,0.04) | 0.01(0.00,0.02) | 0.00(0.00,0.01) | 0.00(0.00,0.00) | 1953.63(686.73,4032.61) | 843.62(309.59,1910.37) | 9.11(3.23,19.28) | 3.60(1.28,8.07) | 1.62(0.91,2.51) | 0.29(0.17,0.47) |
|  | Both | 3.80(1.35,7.98) | 2.39(0.86,5.31) | 0.02(0.01,0.04) | 0.01(0.00,0.02) | 0.00(0.00,0.00) | 0.00(0.00,0.00) | 4245.22(1505.37,8918.27) | 1858.50(670.47,4121.39) | 19.45(6.96,41.43) | 7.84(2.77,17.38) | 2.74(1.78,3.96) | 0.41(0.25,0.65) |
| Tuberculosis | Male | 14.60(11.17,18.90) | 0.31(0.23,0.43) | 1.72(1.27,2.13) | 0.06(0.05,0.08) | 1.29(0.95,1.65) | 0.06(0.05,0.09) | 8650.74(6617.23,11202.93) | 129.38(95.33,177.67) | 932.13(690.71,1154.35) | 29.67(23.07,39.99) | 683.00(501.04,871.82) | 29.21(22.27,40.50) |
|  | Female | 16.84(13.83,20.29) | 0.22(0.17,0.28) | 1.54(1.28,1.80) | 0.04(0.03,0.05) | 1.64(1.36,1.92) | 0.06(0.05,0.08) | 8850.27(7266.87,10663.41) | 80.44(60.19,100.36) | 769.94(640.60,901.57) | 16.48(13.24,20.62) | 813.37(674.23,950.80) | 25.62(20.06,32.47) |
|  | Both | 15.65(13.07,18.48) | 0.27(0.21,0.34) | 1.63(1.36,1.92) | 0.05(0.04,0.06) | 1.46(1.23,1.70) | 0.06(0.05,0.08) | 17501.01(14612.47,20663.08) | 209.82(164.91,267.92) | 1702.07(1413.99,1999.42) | 46.15(38.14,58.10) | 1496.36(1253.94,1736.19) | 54.83(45.53,67.07) |
| Upper respiratory infections | Male | 5.95(1.05,9.34) | 0.19(0.11,0.44) | 0.38(0.06,0.61) | 0.02(0.01,0.04) | 0.20(0.03,0.32) | 0.01(0.01,0.03) | 3524.02(619.42,5535.40) | 79.82(44.90,182.91) | 206.82(31.45,328.22) | 8.93(4.83,21.48) | 103.48(17.34,170.86) | 5.00(2.54,11.89) |
|  | Female | 6.04(1.14,9.24) | 0.13(0.07,0.32) | 0.34(0.05,0.56) | 0.01(0.01,0.03) | 0.09(0.01,0.15) | 0.00(0.00,0.01) | 3176.06(600.66,4856.65) | 46.84(26.46,115.00) | 171.75(22.65,279.26) | 4.96(2.66,12.05) | 46.75(7.13,75.07) | 1.37(0.73,3.60) |
|  | Both | 5.99(1.33,9.09) | 0.16(0.10,0.34) | 0.36(0.06,0.57) | 0.01(0.01,0.03) | 0.15(0.02,0.23) | 0.01(0.00,0.02) | 6700.08(1483.41,10161.23) | 126.67(76.52,267.49) | 378.57(64.64,590.36) | 13.89(8.19,29.64) | 150.24(24.80,237.71) | 6.37(3.59,14.37) |

Table 6: Age-specific burden of DALYs caused by infectious diseases among children in China

| Age groups | | | | | | | | | | | | | |
| --- | --- | --- | --- | --- | --- | --- | --- | --- | --- | --- | --- | --- | --- |
|  |  | ASDR | | | | | | Number of DALYs | | | | | |
|  |  | <5 | | 5-9 | | 10-14 | | <5 | | 5-9 | | 10-14 | |
|  |  | 1990 | 2021 | 1990 | 2021 | 1990 | 2021 | 1990 | 2021 | 1990 | 2021 | 1990 | 2021 |
| Acute hepatitis | Male | 566.24(447.37,727.22) | 12.49(8.26,18.03) | 40.60(33.12,49.22) | 7.34(4.73,11.46) | 27.95(22.10,35.19) | 8.57(5.54,12.74) | 335572.98(265122.37,430968.63) | 5199.67(3440.00,7503.96) | 22023.45(17962.69,26696.00) | 3741.04(2409.15,5836.10) | 14756.81(11667.68,18578.04) | 3941.42(2549.99,5857.28) |
|  | Female | 520.51(410.95,661.00) | 7.85(5.86,10.13) | 34.03(27.39,42.32) | 7.00(4.43,10.66) | 25.16(19.88,31.77) | 8.34(5.45,12.68) | 273490.37(215926.48,347310.58) | 2828.80(2111.76,3651.11) | 17029.82(13707.40,21174.94) | 3138.49(1985.20,4780.37) | 12454.03(9837.68,15722.74) | 3351.21(2189.93,5097.03) |
|  | Both | 544.75(444.09,671.69) | 10.34(7.56,14.06) | 37.45(31.47,44.19) | 7.18(4.70,10.43) | 26.60(21.73,32.58) | 8.46(5.60,12.73) | 609063.35(496522.65,750985.21) | 8028.47(5874.87,10922.63) | 39053.27(32812.66,46079.43) | 6879.52(4498.58,9988.08) | 27210.84(22223.10,33325.83) | 7292.63(4822.69,10975.83) |
| Enteric infections | Male | 5974.52(3803.13,8255.22) | 132.12(92.34,184.25) | 267.27(156.26,399.84) | 43.20(29.01,61.01) | 144.60(91.45,211.41) | 28.38(18.61,41.61) | 3540671.92(2253845.65,4892278.94) | 55004.16(38442.49,76703.93) | 144967.15(84752.22,216869.45) | 22009.71(14778.61,31082.83) | 76350.35(48287.34,111624.84) | 13052.95(8557.95,19139.70) |
|  | Female | 6195.49(4825.03,7689.49) | 133.22(101.60,171.49) | 241.50(134.97,366.76) | 32.68(21.93,46.79) | 136.91(84.71,201.24) | 22.53(14.58,32.93) | 3255304.54(2535222.90,4040302.94) | 48010.69(36615.49,61801.65) | 120840.99(67539.47,183523.38) | 14650.61(9828.60,20972.82) | 67759.28(41924.20,99597.63) | 9057.86(5859.74,13239.78) |
|  | Both | 6078.36(4582.12,7655.33) | 132.63(101.79,169.70) | 254.90(157.91,354.61) | 38.28(25.91,54.28) | 140.88(93.65,201.60) | 25.65(16.90,37.02) | 6795976.46(5123085.90,8559124.44) | 103014.85(79056.65,131804.39) | 265808.14(164667.21,369774.87) | 36660.32(24816.38,51983.74) | 144109.63(95796.46,206223.72) | 22110.81(14568.91,31907.66) |
| HIV/AIDS | Male | 4.64(1.41,7.92) | 45.39(21.08,94.79) | 0.28(0.00,0.68) | 8.09(4.54,14.59) | 0.00(0.00,0.01) | 5.68(3.39,9.49) | 2748.23(835.97,4693.48) | 18894.68(8777.76,39462.86) | 152.64(0.00,367.18) | 4121.67(2311.69,7430.89) | 1.54(0.00,4.34) | 2611.57(1561.15,4365.35) |
|  | Female | 4.65(1.41,7.94) | 46.15(21.37,96.18) | 0.28(0.00,0.67) | 8.23(4.62,14.84) | 0.00(0.00,0.01) | 5.79(3.46,9.68) | 2443.24(739.73,4171.70) | 16632.16(7699.52,34659.54) | 139.08(0.00,336.79) | 3690.48(2069.63,6653.16) | 1.39(0.00,3.93) | 2327.42(1392.73,3893.26) |
|  | Both | 4.64(1.41,7.92) | 45.74(21.21,95.53) | 0.28(0.00,0.68) | 8.16(4.58,14.70) | 0.00(0.00,0.01) | 5.73(3.42,9.58) | 5191.48(1579.24,8858.47) | 35526.83(16477.28,74195.76) | 291.72(0.00,704.73) | 7812.15(4382.91,14082.94) | 2.94(0.00,8.26) | 4938.99(2948.93,8258.61) |
| Lower respiratory infections | Male | 25983.04(21715.09,30388.63) | 1316.67(1035.10,1627.32) | 816.58(697.98,945.15) | 105.96(87.12,129.86) | 310.20(258.00,364.11) | 55.69(45.98,66.62) | 15398296.09(12868986.85,18009173.15) | 548139.46(430917.10,677465.76) | 442909.41(378582.45,512642.38) | 53980.84(44381.89,66152.01) | 163783.48(136220.89,192248.32) | 25611.67(21149.65,30641.20) |
|  | Female | 24002.83(20593.61,28220.94) | 998.28(773.69,1244.86) | 628.27(386.92,755.41) | 79.67(65.18,102.25) | 244.90(136.26,295.41) | 41.25(33.28,53.88) | 12611845.43(10820535.22,14828173.70) | 359755.57(278818.12,448619.80) | 314378.48(193611.75,377998.69) | 35712.31(29219.71,45833.98) | 121210.30(67439.21,146207.22) | 16582.08(13377.38,21659.14) |
|  | Both | 25052.44(21426.16,29270.23) | 1168.94(925.93,1442.06) | 726.22(573.27,834.74) | 93.65(79.06,111.78) | 278.61(219.84,319.92) | 48.95(41.75,59.45) | 28010141.52(23955733.93,32725879.11) | 907895.03(719150.88,1120026.16) | 757287.89(597793.02,870450.20) | 89693.15(75718.17,107050.62) | 284993.78(224883.82,327254.84) | 42193.75(35983.69,51241.10) |
| Neglected tropical diseases and malaria | Male | 290.91(205.26,419.16) | 32.83(21.03,49.93) | 270.33(167.35,443.46) | 27.15(18.27,40.75) | 244.52(147.69,364.57) | 35.31(21.95,54.54) | 172400.63(121644.05,248407.13) | 13669.31(8755.45,20788.23) | 146623.11(90767.69,240527.89) | 13829.38(9305.25,20761.31) | 129105.26(77979.43,192487.71) | 16240.38(10094.06,25084.28) |
|  | Female | 221.36(163.35,303.62) | 29.41(20.04,42.22) | 215.87(131.87,327.58) | 26.30(17.90,37.03) | 214.78(124.16,330.49) | 33.97(22.45,50.57) | 116308.70(85828.05,159531.97) | 10596.95(7221.97,15215.93) | 108017.82(65986.04,163915.45) | 11789.17(8025.30,16598.14) | 106303.70(61449.14,163568.44) | 13654.72(9024.82,20328.10) |
|  | Both | 258.22(183.61,361.84) | 31.24(20.72,44.74) | 244.19(150.70,384.31) | 26.75(18.33,37.95) | 230.13(135.25,348.32) | 34.68(22.19,51.42) | 288709.32(205285.62,404560.91) | 24266.26(16090.68,34746.58) | 254640.92(157148.87,400746.55) | 25618.55(17551.58,36348.23) | 235408.97(138349.18,356302.58) | 29895.11(19122.57,44317.27) |
| Other infectious diseases | Male | 6166.54(3980.01,8745.11) | 427.22(293.49,631.81) | 676.72(475.78,1058.94) | 90.72(73.15,118.65) | 285.37(226.88,389.03) | 56.30(45.70,70.65) | 3654471.22(2358665.39,5182601.91) | 177856.23(122181.21,263026.31) | 367046.50(258061.76,574360.17) | 46215.39(37264.12,60443.45) | 150674.00(119788.54,205407.42) | 25894.62(21020.39,32493.17) |
|  | Female | 5394.70(3472.49,8454.02) | 361.84(245.71,544.16) | 612.50(388.80,1035.77) | 84.26(65.46,113.33) | 234.11(178.71,332.29) | 53.10(43.10,65.66) | 2834543.83(1824554.20,4442008.89) | 130397.17(88546.95,196104.10) | 306488.02(194551.74,518286.71) | 37769.43(29343.14,50803.34) | 115866.40(88450.54,164461.59) | 21344.85(17327.16,26396.49) |
|  | Both | 5803.82(3835.76,8399.26) | 396.88(279.82,582.52) | 645.90(440.55,1046.96) | 87.69(70.09,114.38) | 260.57(208.09,365.68) | 54.81(45.13,66.56) | 6489015.04(4288614.46,9390875.66) | 308253.40(217333.70,452436.83) | 673534.52(459398.57,1091753.30) | 83984.83(67127.15,109538.51) | 266540.40(212855.94,374061.86) | 47239.46(38895.60,57368.14) |
| Otitis media | Male | 43.10(24.90,70.77) | 42.53(24.31,69.81) | 55.88(32.28,89.97) | 50.33(29.14,80.11) | 50.41(28.49,80.02) | 41.05(23.12,67.61) | 25539.35(14753.88,41939.03) | 17705.11(10120.07,29061.21) | 30310.18(17509.14,48797.48) | 25637.47(14847.13,40810.25) | 26614.68(15043.97,42247.83) | 18878.86(10633.48,31095.37) |
|  | Female | 41.79(23.42,70.23) | 40.46(22.44,67.13) | 47.91(27.14,76.82) | 42.66(23.80,68.35) | 40.67(23.10,63.88) | 32.93(17.79,53.27) | 21958.47(12307.31,36898.53) | 14582.24(8086.32,24192.45) | 23971.14(13581.03,38439.90) | 19123.38(10667.19,30638.95) | 20128.66(11434.91,31617.48) | 13237.97(7150.65,21416.53) |
|  | Both | 42.48(24.30,70.24) | 41.57(23.58,68.49) | 52.05(29.65,82.98) | 46.74(26.49,73.76) | 45.70(25.75,71.86) | 37.26(20.68,60.36) | 47497.82(27166.90,78535.33) | 32287.35(18310.61,53196.79) | 54281.32(30918.85,86524.94) | 44760.85(25372.05,70638.98) | 46743.33(26338.07,73509.62) | 32116.83(17828.91,52024.14) |
| Sexually transmitted infections excluding HIV | Male | 347.58(123.03,739.96) | 219.08(76.36,477.54) | 1.72(0.69,3.56) | 0.70(0.25,1.58) | 1.40(0.76,2.47) | 1.01(0.47,1.94) | 205985.89(72913.19,438520.74) | 91202.76(31789.49,198803.27) | 931.18(374.30,1928.74) | 355.56(126.70,805.66) | 737.93(399.78,1304.07) | 462.50(213.89,890.00) |
|  | Female | 334.23(117.54,689.80) | 210.38(77.21,476.38) | 1.64(0.66,3.34) | 0.67(0.24,1.50) | 0.84(0.58,1.22) | 0.46(0.27,0.76) | 175614.27(61761.74,362443.69) | 75815.96(27825.84,171674.33) | 823.04(329.33,1670.95) | 301.88(109.72,672.90) | 413.96(286.40,604.79) | 186.10(108.65,304.51) |
|  | Both | 341.31(121.07,716.95) | 215.04(77.59,476.83) | 1.68(0.68,3.44) | 0.69(0.25,1.51) | 1.13(0.70,1.79) | 0.75(0.41,1.34) | 381600.16(135362.85,801598.06) | 167018.72(60259.58,370344.25) | 1754.23(713.59,3585.96) | 657.44(237.31,1448.47) | 1151.89(714.54,1832.61) | 648.60(349.79,1156.00) |
| Tuberculosis | Male | 1344.97(1037.48,1732.30) | 37.99(30.06,48.33) | 158.58(121.32,193.81) | 8.55(6.45,11.31) | 104.57(77.38,131.86) | 5.90(4.62,7.71) | 797065.56(614841.42,1026610.50) | 15814.56(12514.36,20118.74) | 86013.88(65805.45,105123.63) | 4354.98(3285.78,5762.43) | 55214.85(40858.24,69623.77) | 2712.65(2123.09,3546.60) |
|  | Female | 1549.20(1270.82,1859.12) | 31.28(24.19,38.24) | 151.39(125.94,179.52) | 6.98(5.14,9.45) | 134.19(112.14,156.39) | 5.97(4.73,7.36) | 813998.53(667727.69,976839.05) | 11272.60(8717.69,13782.02) | 75752.24(63017.29,89831.06) | 3127.38(2303.57,4235.24) | 66416.50(55502.64,77404.59) | 2399.83(1901.17,2958.92) |
|  | Both | 1440.95(1210.53,1701.25) | 34.88(28.31,43.22) | 155.13(130.03,181.84) | 7.81(5.95,10.26) | 118.91(99.83,138.38) | 5.93(4.93,7.03) | 1611064.09(1353446.11,1902099.23) | 27087.16(21988.61,33569.62) | 161766.12(135596.63,189615.65) | 7482.36(5699.27,9826.88) | 121631.35(102113.98,141549.42) | 5112.48(4251.52,6062.83) |
| Upper respiratory infections | Male | 624.72(191.65,926.96) | 113.25(69.99,176.09) | 103.78(64.63,158.26) | 72.94(40.46,122.28) | 67.33(40.10,104.31) | 52.55(27.66,87.70) | 370225.00(113576.02,549341.28) | 47147.75(29138.24,73306.28) | 56288.90(35055.08,85839.81) | 37158.86(20609.33,62290.65) | 35551.76(21172.26,55075.97) | 24170.35(12719.85,40337.43) |
|  | Female | 627.90(185.04,911.15) | 104.50(64.13,168.23) | 98.37(59.59,152.59) | 70.63(38.76,118.08) | 57.89(32.54,94.06) | 50.59(26.52,85.96) | 329920.00(97226.05,478748.94) | 37657.69(23111.48,60624.63) | 49221.36(29819.29,76355.62) | 31662.47(17377.10,52933.69) | 28652.16(16104.88,46555.17) | 20337.41(10659.18,34555.58) |
|  | Both | 626.21(215.49,911.32) | 109.19(67.20,172.14) | 101.18(63.32,153.44) | 71.86(39.67,120.11) | 62.77(37.09,98.91) | 51.64(27.14,86.21) | 700145.00(240933.74,1018912.58) | 84805.45(52190.38,133694.93) | 105510.26(66032.40,160006.08) | 68821.33(37992.97,115027.62) | 64203.93(37945.14,101173.87) | 44507.76(23395.37,74306.44) |
